# Bioinformatics-aided identification, characterization and applications of mushroom linalool synthases

**DOI:** 10.1038/s42003-021-01715-z

**Published:** 2021-02-17

**Authors:** Congqiang Zhang, Xixian Chen, Raphael Tze Chuen Lee, Rehka T, Sebastian Maurer-Stroh, Martin Rühl

**Affiliations:** 1 Singapore Institute of Food and Biotechnology Innovation (SIFBI), Agency for Science, Technology and Research (A*STAR), Singapore, Singapore; 2 Bioinformatics Institute (BII), Agency for Science Technology and Research (A*STAR), Singapore, Singapore; 3 Department of Biological Sciences (DBS), National University of Singapore (NUS), Singapore, Singapore; 4 Institute of Food Chemistry and Food Biotechnology, Justus Liebig University Giessen, Giessen, Germany

**Keywords:** Metabolic engineering, Industrial microbiology, Biocatalysis, Biosynthesis

## Abstract

Enzymes empower chemical industries and are the keystone for metabolic engineering. For example, linalool synthases are indispensable for the biosynthesis of linalool, an important fragrance used in 60–80% cosmetic and personal care products. However, plant linalool synthases have low activities while expressed in microbes. Aided by bioinformatics analysis, four linalool/nerolidol synthases (LNSs) from various Agaricomycetes were accurately predicted and validated experimentally. Furthermore, we discovered a linalool synthase (Ap.LS) with exceptionally high levels of selectivity and activity from Agrocybe pediades, ideal for linalool bioproduction. It effectively converted glucose into enantiopure (R)-linalool in Escherichia coli, 44-fold and 287-fold more efficient than its bacterial and plant counterparts, respectively. Phylogenetic analysis indicated the divergent evolution paths for plant, bacterial and fungal linalool synthases. More critically, structural comparison provided catalytic insights into Ap.LS superior specificity and activity, and mutational experiments validated the key residues responsible for the specificity.

## Abstract (web summary)

Zhang et al. identified four linalool/nerolidol synthases from fungi using bioinformatics and phylogenetic analysis and validated their functions with in vitro and in vivo methods. One of them is a rare and highly specific monoterpene synthase and responsible for impressive titres of the commercially sought-after fragrance (R)-linalool.

## Introduction

Nature is the best inventor and breeds versatile enzymes. Among various enzymes, terpene synthases (TPSs) represent a unique class of biocatalysts with fascinating capabilities (e.g., introduction of carbon–carbon bonds, facilitation of cyclization, and rearrangement of terpenes)^1^. Terpene synthases are pivotal for the biosynthesis and diversity of terpenoids (>80,000 different molecules), which constitute the largest group of natural products^1^. Terpenoids have wide applications, including pharmaceuticals, nutraceuticals, flavorings, fragrances, and biofuels^2,3^. However, the biosynthesis of most terpenoids has yet to achieve high titers and yields that are vital for commercial production. One major obstacle is that currently identified TPSs have low activities and/or low selectivities^2,4^.

**Fig. 1:**
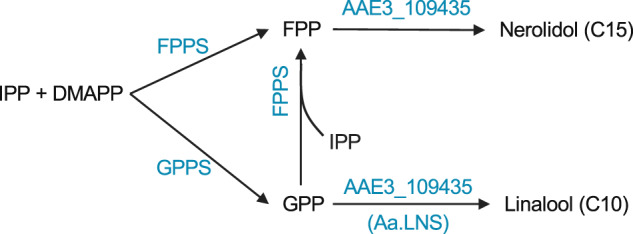
The brief biosynthetic pathway of linalool and nerolidol. Linalool is a monoterpene alcohol (C10), produced from geranyl pyrophosphate (GPP). Nerolidol is a sesquiterpene alcohol (C15), produced from farnesyl pyrophosphate (FPP).

An example is linalool, a naturally occurring monoterpene alcohol (C10) found in several flowers such as lavender^5^. With a pleasant floral smell, linalool is an important fragrance ingredient widely used in food, beverage, and many personal care products (perfumes, body lotions, etc.). Natural linalool has two stereoisomers with different smells, (*S*)-linalool and (*R*)-linalool. (*S*)-linalool is floral, citrus, and petitgrain-like (odor threshold 7.4 ppb) and (*R*)-linalool is woody and lavender-like (odor threshold 0.8 ppb) and present also in sweet basil^6^. Natural linalool has higher enantiopurity, thus superior to synthetic linalool racemates in applications such as high-end perfumes and cosmetics. In 2018, the world consumption of linalool surpassed 11,000 metric tons and its global market is projected to reach 12.3 billion US$ in 2024^7^. Despite great commercial interests, the biosynthesis of linalool has only achieved limited success (mg/L scale)^8,9^. This contrasts with the rapidly growing demand for natural linalool. The plant linalool synthases (converting geranyl pyrophosphate (GPP) into linalool, Fig. 1) are relatively abundant, yet proven to have low activities when expressed in microbial hosts (e.g., yeasts and *Escherichia coli*)^10^. Recently, a bacterial bifunctional linalool/nerolidol synthase (LNS) has been identified and characterized^11^. However, it produces more nerolidol (a sesquiterpene alcohol, the product of farnesyl pyrophosphate (FPP), Fig. 1) than linalool when expressed in microbes. This is because, unlike plants that have special compartments (e.g., plastids) where GPP synthases (GPPSs) are localized^12^, wild-type microbes have neither specialized organelles nor dedicated GPPSs. Rather, GPP is merely an intermediate compound of FPP synthases (e.g., ispA of *E. coli*, ERG20 of *Saccharomyces cerevisiae*) in microbes (Fig. 1). Hence, FPP is more abundant than GPP in the cytosol^13^. As such, a specific and more active linalool synthase is desired for microbial linalool production. Besides plants and bacteria, fungi such as the agaric mushroom *Agrocybe aegerita* (recently renamed into *Cyclocybe aegerita*), are also known to produce linalool and several other monoterpenes (α-pinene, *p*-cymene, limonene, and β-ocimene)^14^. However, fungal linalool synthases have not been identified until very recently. In our previous study^9^, we described the bifunctional role of an LNS from *A. aegerita* that was used to produce linalool or nerolidol in *E. coli* but did not elaborate on the method of identification or its kinetic characterization.

Here, guided by bioinformatics predictions and experimental validation, we identified four bifunctional LNSs and a specific linalool synthase (no sesquiterpene activity) from four fungal species. We characterized the kinetic parameters of the LNS from *A. aegerita* and the linalool synthase from *Agrocybe pediades*, both saprobic mushrooms. Phylogenetic analysis indicated clear divergence among the 35 plant enzymes (4 nerolidol synthases, 9 linalool synthases, and 22 LNSs), 1 bacterial LNS (D5SL78), and 9 fungal enzymes (LNSs and linalool synthases). Furthermore, in vitro and in vivo activities and structural and functional comparison were carried out for the plant, bacterial, and fungal linalool synthases. It was found that the fungal linalool synthase from *A. pediades* is highly active in *E. coli* and has an exceptional selectivity, thus ideal for linalool biosynthesis in metabolic engineering and other biocatalytic processes. On top of structural analysis, further mutation experiments identified the key residues responsible for the specificity of the fungal linalool synthase, as compared to fungal LNSs. The knowledge is essential for the design of artificial enzymes and interconversion between monoterpene synthases and sesquiterpene synthases.

## Results and discussion

Plant metabolites are well recognized and explored. In contrast, the biosynthetic capability of fungi is relatively undervalued. Fungi (>3 million species) have enormous diversity and are rich in biocatalysts and secondary metabolites^15–17^. In particular, fungi produce a large portfolio of volatile terpenoids, including sesquiterpenes (e.g., β-caryophyllene and cubenol^18^) and monoterpenes (linalool^14^, limonene^14^, and 1,8-cineole^19^). To our knowledge, to date, there are only a few fungal monoterpene synthases reported in the literature (Hyp3, a 1,8-cineole synthase^19^ and the very recently identified PpSTS25, a bifunctional myrcene/linalool synthase^20^). Here we address this gap and specifically explored fungal linalool synthases.

### Identification of linalool synthase in agaric mushroom

As *A. aegerita* is known to produce linalool and its genome has been recently sequenced^21^, we tried to identify the linalool synthase in its genome. In our previous study, we identified 11 putative sesquiterpene synthases and 9 of them are functional and produced various sesquiterpenes but not linalool^3^. A re-evaluation of the raw genomic data led to an additional putative TPS sequence (AAE3_109435, accession number MN954676). The gene exists in the Illumina sequencing data, whereas it is absent in the PacBio results. PCR amplification of the AAE3_109435 with subsequent sequencing confirmed the presence of the gene in the genome (Supplementary Fig. S1).

**Fig. 2:**
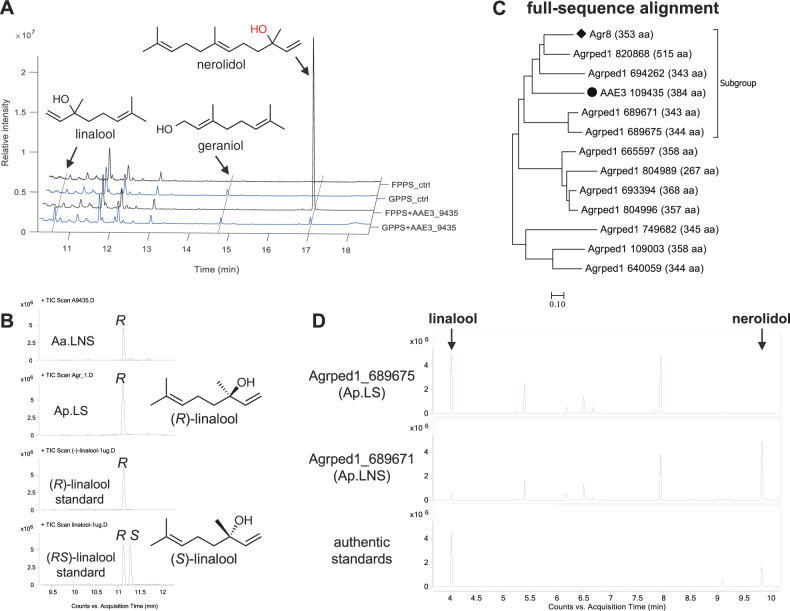
Characterization of fungal linalool and nerolidol synthases (LNSs) and a fungal linalool synthase in *E. coli*. **A** GC-MS chromatograms of cultural supernatants of strains GPPS+9435, FPPS+9435 (co-expressing AAE3_109435 and GPP or FPP synthase), and the control strains GPPS_ctrl and FPPS_ctrl (expressing only GPP or FPP synthase). **B** Chiral separation of linalool produced by Aa.LNS and Ap.LS (Agrped1_689675) expressing strains. **C** Phylogenetic tree based on full-sequence alignment of Aa.LNS and terpene synthases (TPSs) in *A. pediades*. AAE3_109435 (a LNS) is marked with black circles (●), Agr8 (a γ-muurolene/β-cadinene synthase) with black diamonds (◆). **D** GC-MS chromatograms of the supernatant of *E. coli* clones producing Agrped1_689671 and Agrped1_689675.

The subsequent expression of AAE3_109435 in a GPP-accumulating *E. coli* that co-expressed the native enzymes DXS, IDI, and ispA_S80F mutant (GPPS). The resulting strain (GPPS+9435) produced the acyclic monoterpene linalool as the main product and small amount of nerolidol (Fig. 2 and mass spectra in Supplementary Fig. S2). Geraniol was detected in GPPS+9435 as well as in its control strain (GPPS_ctrl), indicating that geraniol is not the product of AAE3_109435 but instead that of native *E. coli* enzymes (such as PhoA, a phosphatase, and NudB, a Nudix hydrolase^22^). Furthermore, AAE3_109435 was expressed in the *E. coli* strain that accumulates FPP by overexpressing DXS and IDI (without ispA_S80F), and the strain was named ‘FPPS+9435.’ Its main product (>96% regarding the peak area of all the detected terpenes) was nerolidol and only traces of linalool could be detected (Fig. 2A). The control strain (FPPS_ctrl) with an empty vector produced neither linalool nor nerolidol. The results clearly proved that the TPS coded by AAE3_109435 is a bifunctional LNS, which is able to convert FPP into nerolidol and GPP into linalool. Accordingly, it is named Aa.LNS. Furthermore, the linalool produced by Aa.LNS is mainly (*R)*-linalool (95% ee, Fig. 2B).

### Bioinformatics prediction of other fungal LNSs

Aa.LNS was used to probe other potential fungal LNSs. The first focus was on *A. pediades*, another sequenced fungal species of the genus *Agrocybe*. Linalool was detected in the headspace of *A. pediades* cultures grown in malt extract medium in our laboratory. A blast search of Aa.LNS against the *A. pediades* genome using the online tool of the Joint Genome Institute (https://genome.jgi.doe.gov/Agrped1/Agrped1.home.html) resulted in 11 TPS homologs (Fig. 2C).

To improve the prediction confidence, we combined two strategies: (1) full-sequence alignment (Fig. 2C and protein sequence in Supplementary Data 1) and (2) comparison of predicted active sites (Supplementary Tables S1 and S2, the results were analyzed with 4LXW (Epi-isozizaene synthase from *Streptomyces coelicolor*) and 5NX5 (PDB ID, the bacterial linalool synthase from *Streptomyces clavuligerus*, or Sc.LNS) as templates). Here 4LXW and 5NX5 are chosen based on the two criteria: (1) the higher sequence similarity to Aa.LNS and (2) the availability of large ligands (either substrate or product analogs) in the crystal structures, which can facilitate the identification of the active-site residues. Four TPS homologs (Agrped1_820868, Agrped1_694262, Agrped1_689671, and Agrped1_689675) were found to be closely related to Aa.LNS (Fig. 2C). It was hypothesized that the two enzymes Agrped1_820868 and Agrped1_694262 are more similar to Agr8 (a γ-muurolene/β-cadinene synthase)^3^ as all three enzymes share almost identical active sites based on our algorithm (Supplementary Table S1). Hence, we studied Agrped1_689671 and Agrped1_689675. As predicted, the strain expressing Agrped1_689671 produced linalool and nerolidol (renamed Ap.LNS) similar to Aa.LNS (Fig. 2D). Surprisingly, linalool was the sole terpene product of the *E. coli* clone expressing Agrped1_689675, indicating that it is a monofunctional linalool synthase (renamed Ap.LS). This attributes to the high specificity of Ap.LS, which is underpinned by the fact that FPP is more abundant in microbial cells than GPP^13^. The high specificity of Ap.LS is very interesting, possibly due to steric hindrance of some amino acid residues surrounding the binding pocket to the larger substrate FPP. And we will look into this in the later section of this article. In addition, Ap.LS produced the enantiopure (*R*)-linalool (Fig. 2B).

Next, we asked whether we could use Ap.LS to probe other fungal linalool synthases. We carried out a UniProt BLAST search of Ap.LS and collected those hits with the highest alignment score (score >700, Supplementary Fig. S3): three from *Galerina marginata* (Galma_223690, UniProt ID A0A067THX9; Galma_63556, A0A067T8I8; Galma_266794, A0A067T571); two from *Hypholoma sublateritium* (Hypsu1_148365, A0A0D2NH86; Hypsu1_148385, A0A0D2NA50), and one from *Hebeloma cylindrosporum* (M413_27416, A0A0C2YLE7).

**Fig. 3:**
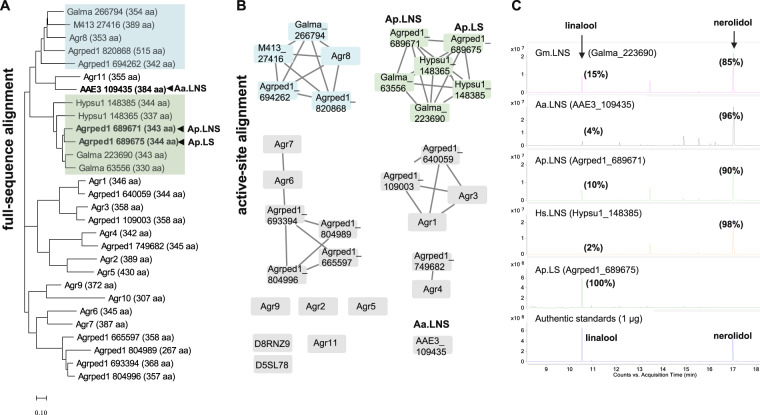
Sequence analysis of fungal TPS homologs. **A** Phylogenetic tree based on full-sequence alignment consisting of 12 TPS homologs from *A. aegerita* (Aa.LNS and Agr), 11 from *A. pediades* (Ap.LS, Ap.LNS and Agrped1), 3 from *Galerina marginata* (Galma), 2 from *Hypholoma sublateritium* (Hypsu1), and 1 from *Hebeloma cylindrosporum* (M413_27416). **B** Sequence similarity network (SSN) built on the predicted active sites (Supplementary Table S1). Agr10 is excluded as it shares limited similarity to the templates (PDB ID: 4LXM and 5NX5). Instead, D5SL78 from *Streptomyces clavuligerus* and D8RNZ9 from *Selaginella moellendorffii* (Spikemoss) are included. The rest of the candidates are the same as those of full-sequence alignment. LNS and muurolene/cadinene synthase groups are highlighted in green and blue, respectively. **C** Experimental validation of the predicted fungal TPS homologs. Ap.LS produced exclusively linalool; the other four produced both nerolidol (85–98%) and linalool (15–2%). Here percentage (%) refers to the peak area ratios of linalool or nerolidol to the sum of the peak areas of both substances present in each chromatogram.

Four of them (Galma_223690, Galma_63556, Hypsu1_148365, and Hypsu1_148385) clustered into a branch or subgroup with Ap.LS and Ap.LNS in both analyses using full-sequence and active-site alignment (Fig. 3A, B and protein sequence in Supplementary Data 1). Overall, the six homologs share >90% identity of active-site residues (Supplementary Tables S1 and S2). Particularly, the active sites of Hypsu1_148385 and Galma_223690 show 95% (36/38) and 97–100% (37–38/38) identity with that of Ap.LS and Ap.LNS, respectively, indicating that they are potential fungal LNSs. The other two amino acid sequences (Galma_266794 and M413_27416) were more closely related to Agr8, with as high as 95% active-site identity (Supplementary Table S1). Thus it was hypothesized that they were more likely to produce 1,10 cyclization products of FPP, e.g., muurolene, cadinene. This hypothesis was validated by the expression of Galma_223690, Hypsu1_148385, or Galma_266794 in the FPP-accumulating *E. coli*. As predicted, Galma_223690 and Hypsu1_148385 were found to be bifunctional LNSs (Fig. 3C), thus renamed Gm.LNS and Hs.LNS, respectively. Moreover, like Agr8, the strain expressing Galma_266794 produced germacrene D (1,10 cyclization) as the main product and a few minor products (γ-muurolene and (+)-δ-cadinene, Supplementary Fig. S4), thus validating our hypothesis.

Here the synergistic use of BLAST search, full-sequence alignment, and active-site alignment was explored. As such, we achieved a relatively high predictability of hunting for biocatalysts of the same function (e.g., linalool synthases). Such a method can be potentially applied for the identification of all kinds of enzymes. BLAST search helps with identifying overall similar enzymes that form our initial screening candidates. Full-sequence alignment and phylogenetic tree facilitate the classification of enzymes. Different classes often have distinct catalytic functions (e.g., different cyclization positions). Active-site prediction further supplements the prediction with two main roles. One is to filter out those enzyme candidates with incomplete binding pockets (e.g., Agr11 is missing the NSE triad, Supplementary Tables S1 and S2). The other is to complement the enzyme classification of full-sequence alignment. This is based on the hypothesis that enzymes of the same function may have overall low similarity but more conserved active sites. For example, the Galma_266794 shares comparable full-sequence similarities with Ap.LS (61%) and Agr8 (62%); however, it has much higher active-site identity with Agr8 (95%) than with Ap.LS (71%, Supplementary Table S1). Indeed, the products of Galma_266794 are similar to that of Agr8.

### Purification and characterization of fungal linalool synthases and LNSs

To date, a number of plant linalool synthases and LNSs have been identified. However, only one bacterial LNS from *S. clavuligerus* was recently identified^11^, which only shares 15.2% identity with Ap.LS (Supplementary Fig. S5). With the fungal enzymes studied in this work, linalool synthases and LNSs in three kingdoms have been identified. Next, we sought to compare their catalytic activities and mechanisms by in vitro, in vivo assays, sequence alignments, and three-dimensional (3D) structural models.

**Fig. 4:**
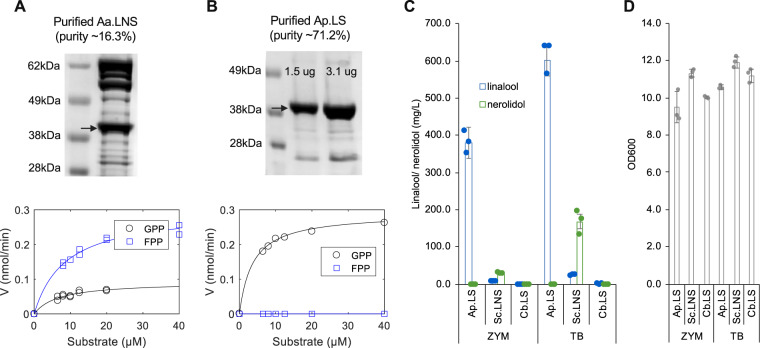
In vitro characterization of Aa.LNS and Ap.LS and in vivo comparison of linalool synthases from different kingdoms in linalool bioproduction. **A** SDS-PAGE gel image of purified Aa.LNS and its kinetic analysis. **B** Protein gel of purified Ap.LS and its kinetic analysis (no product was observed for FPP). **C** In vivo linalool yield comparison of linalool synthases from a fungus, a bacterium, and a plant (error bars, mean ± s.d., *n* = 3). **D** The OD_600_ of different strains in Fig. 3C. Ap.LS, Sc.LNS, and Cb.LS from *Clarkia breweri* (Q96376) were selected as the representative of kingdoms fungi, bacteria, and plantae, respectively.

Protein purification is the prerequisite to study the in vitro kinetics of the fungal enzymes. Though all the five bacterial strains expressing fungal enzymes produced linalool, their expression levels in *E. coli* were largely different. Aa.LNS had the highest expression level, followed by Gm.LNS, Ap.LNS, and Ap.LS. The expression of Hs.LNS (Hypsu_148385) was so low that it was not detectable in a protein gel (Supplementary Fig. S6A). As Aa.LNS had the highest expression level and Ap.LS is the only specific monoterpene synthase, they were chosen as the representatives of fungal LNS and linalool synthase for further studies. However, none of them was soluble based on solubility analysis with B-PER II reagent (Thermo Scientific™) (Supplementary Fig. S6A). Many approaches were tested but failed to improve their solubility (such as abiotic condition optimization: lowering incubation temperature, tuning inducer dosages, media additives, and protein fusion). Refolding of insoluble fraction could be another solution which we did not test because it is very time-consuming to optimize the best conditions. The N-terminal fusion of Aa.LNS with a maltose-binding protein or thioredoxin did not help (Supplementary Fig. S6B). Different chaperone systems (DnaK-dnaJ, GroES-GroEL) and trigger factor (TF) in *E. coli* were further tested. It was found that TF chaperone could slightly improve the solubility of the synthases. With the optimal condition (3.3 mM arabinose to induce TF chaperone and 0.1 mM IPTG to induce Aa.LNS, Supplementary Fig. S7) and further separation by size exclusion chromatography, we managed to purify enough soluble Aa.LNS for in vitro characterization. Yet its purity was quite low with ~16.3% (Fig. 4A and full gel image at Supplementary Fig. S10). In contrast, relatively high purity of soluble Ap.LS (~71.2%) was obtained with the same experimental conditions (Fig. 4B and full gel image at Supplementary Fig. S11). Consistent with the *E. coli* cultures producing the respective synthase, purified enzymes reconfirmed that Aa.LNS can use FPP and GPP to produce nerolidol and linalool, respectively. However, Ap.LS was only active with GPP but not with FPP (Fig. 4). Based on the data in Fig. 4A, B (Supplementary Data 2 and 3), *K*_m_ and *k*_cat_ values of Ap.LS and Aa.LNS were calculated. The *K*_m_ and *k*_cat_ values of Aa.LNS for FPP were 9.0 ± 2.3 μM and 3.3 ± 0.3 min^−1^, respectively, and slightly lower for GPP with 6.7 ± 4.6 μM and 0.5 ± 0.1 min^−1^, respectively (Table 1). The *K*_m_ value of Ap.LS for GPP with 3.8 ± 0.7 μM was slightly lower than that for Aa.LNS, whereas *k*_cat_ was much higher with 6 ± 0.3 min^−1^. To compare the catalytic efficiencies among the known linalool synthases and LNSs, *k*_cat_/*K*_m_ value of Ap.LS was the highest, which is about 21-fold, 29-fold, 3-fold, and 4-fold higher than that of Aa.LNS, La.LS (Q2XSC5) from *Lavandula angustifolia* (Lavender)^5^, Ma.LS (Q8H2B4) from *Mentha aquatica*^23^, and of the bacterial Sc.LNS^11^, respectively. As for Aa.LNS as a nerolidol synthase, although the *k*_cat_ value of Aa.LNS for FPP was more than five times lower compared to the bacterial one, the *K*_m_ value was similar to that of the bacterial Sc.LNS^11^ and about half as that of Zm.LNS from *Zea mays* (Maize) (Table 1)^24^.

**Table 1:** Comparison of kinetic parameters for linalool synthases and LNSs from fungi, plant, and bacteria.

	Sesquiterpene synthase			Monoterpene synthase				
Enzymes	Aa.LNS	Zm.LNS	Sc.LNS	Ap.LS	Aa.LNS	La.LS	Ma.LS	Sc.LNS
Accession no.	/	Q29VN2	D5SL78	/	/	Q2XSC5	Q8H2B4	D5SL78
Product	Nerolidol	Nerolidol	Nerolidol	(*R*)-linalool	(*R*)-linalool	(*R*)-linalool	(*R*)-linalool	(*R*)-linalool
Organism	*Agrocybe aegerita*	*Zea mays*	*Streptomyces clavuligerus*	*Agrocybe pediades*	*Agrocybe aegerita*	*Lavandula angustifolia*	*Mentha aquatica*	*Streptomyces clavuligerus*
Kingdom	Fungi	Plantae	Bacteria	Fungi	Fungi	Plantae	Plantae	Bacteria
*K*_m_ (μM)	9.0 ± 2.3	18.0 ± 7.8	9.6 ± 0.8	3.8 ± 0.7	6.7 ± 4.6	42.7	25 ± 6	12.9 ± 1.3
*k*_cat_ (min^−1^)	3.3 ± 0.3	—	18.7 ± 0.7	6.0 ± 0.3	0.5 ± 0.1	2.34	14.4 ± 1.2	5.0 ± 0.1
*k*_cat_/*K*_m_ (min^−1^ μM^−1^)	0.4	—	1.9	1.6	0.1	0.05	0.6	0.4
Reference	This study	^24^	^11^	This study	This study	^5^	^23^	^11^

### In vivo activity comparison of linalool synthases and LNSs from three kingdoms and applications in linalool production

Due to potential issues such as poor expression and solubility when expressed in cells and the localization difference (cytosolic/membrane bound in vivo versus a one-pot aqueous reaction), the advantages of in vitro enzyme kinetics (Table 1) may not be readily transferable into cellular applications, such as metabolic engineering, where in vivo activities are more critical than in vitro ones. To test the best candidate for microbial linalool production, our previously engineered *E. coli* strain was used to compare linalool synthases from three kingdoms: Ap.LS, Sc.LNS, and Cb.LS from *Clarkia breweri* (Q96376) as representatives for fungi, bacteria, and plantae, respectively. They were separately cloned into pET-11a vector (Novagen). Together with a p15A vector carrying the whole mevalonate pathway genes^2^, the bacterial strains grown in ZYM media produced linalool at 381.2, 8.7 and 1.3 mg/L for fungal, bacterial, and plant linalool synthases, respectively (Fig. 4C). The linalool yield using Ap.LS (fungal) is about 44- and 287-fold as high as that using Sc.LNS (bacterial) and Cb.LS (plant), respectively. As the bacterial densities for different strains are similar, around 10–12 (Fig. 4D), the high yield of linalool in the Ap.LS strain was because of its relatively high in vivo activity (here we refer to the total activity that is the result of both the specific activity and the amount of active enzyme) but not of biomass. A previous study also supported that the bacterial Sc.LNS is better than plant linalool synthases in linalool production in terrific broth (TB) media^10^. In the same TB media, the linalool titers reached 601.2 mg/L for Ap.LS stain, about 65% higher than previously reported using Sc.LNS^10^. Our study demonstrated that fungal Ap.LS is even superior to the bacterial one, in both activity and selectivity. Although Sc.LNS has a higher activity than plant Cb.LS, it prefers FPP (lower *K*_m_ and higher *k*_cat_) to GPP as the substrate^11^. Therefore, Sc.LNS produced a larger amount of nerolidol than linalool in *E. coli* whose cytosol contained both FPP and GPP; in contrast, Ap.LS produced 100% linalool.

High activity contributes to high titers, rates, and yields (TRYs) of linalool production and low manufacturing cost. High specificity would greatly simplify the downstream purification process and further reduce the overall production cost. The superior activity and selectivity of Ap.LS make it more suitable for microbial production of linalool than its plant and bacterial counterparts. Thus, this study sets up a foundation for future works of linalool bioproduction that is greener, safer, sustainable, and of exceptional enantiopurity ((*R*)-linalool), as compared to chemical synthesis. However, to translate into commercial applications, more studies are required to further improve the linalool TRYs and to overcome the toxicity issue of linalool, which can be addressed by genetic engineering (e.g., metabolic engineering, efflux transporter engineering^25^), directed evolution, and bioprocess developments (e.g., in situ product remove fermentation using suitable liquid solvents and/or solid absorbents)^26^.

### Structural comparison of linalool synthases and LNSs from three kingdoms (plants, fungi, and bacteria)

**Fig. 5:**
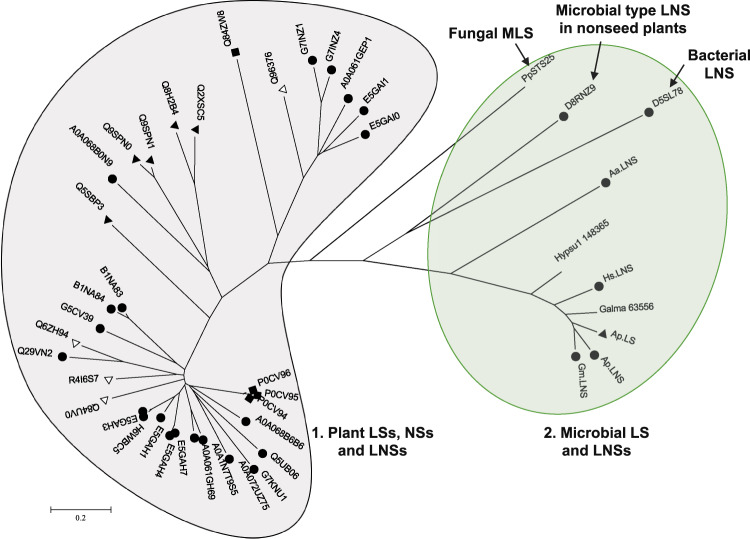
Phylogenetic tree of LNSs and linalool synthases in plants, fungi, and bacteria. Two major clades, plant and microbial, are clearly segmented. (*R*)-linalool synthases are marked with black triangles (▲), (*S*)-linalool synthases with white triangles (△), LNSs with black circles (●), and nerolidol synthases with black rectangles (■). PpSTS25 is a myrcene/linalool synthase from *Postia placenta*^20^. For plant and bacterial enzymes, UniProt accession number are used. D8RNZ9, more similar to the bacterial LNS than plant ones, was a LNS isolated from the nonseed plant *Selaginella moellendorffii* (Spikemoss). Details of all the enzymes are in Supplementary Table S3.

Next, we generated a phylogenetic tree with 35 plant enzymes (including 4 nerolidol synthases, 9 linalool synthases and 22 LNSs), 1 bacterial LNS (D5SL78), and 9 fungal enzymes (Supplementary Table S3). The enzymes were clearly separated into two major clades (one is plant, clade 1, and the other is microbial, clade 2, Fig. 5). The bacterial LNS was closer to the fungal ones, in clade 2. Specifically, the sequence identity among fungal, bacterial, and plant LNSs or linalool synthases are only 8–15%, which includes those metal-binding sites (Supplementary Fig. S5). Overall, plant linalool synthases/LNSs are larger with 500–900 amino acids than microbial ones with 300–400 amino acids. One particular enzyme D8RNZ9, which is a LNS isolated from the nonseed plant *Selaginella moellendorffii* (Spikemoss)^27^, is more closely related to the bacterial LNS than plant LNSs. It was hypothesized that it could stem from horizontal gene transfer from microbes to plants or that seed plants lost these LNS enzymes during evolution from nonseed plants^27^. The phylogenetic tree indicates the evolutionary divergence of fungal, bacterial, and plant linalool synthases and LNSs.

**Fig. 6:**
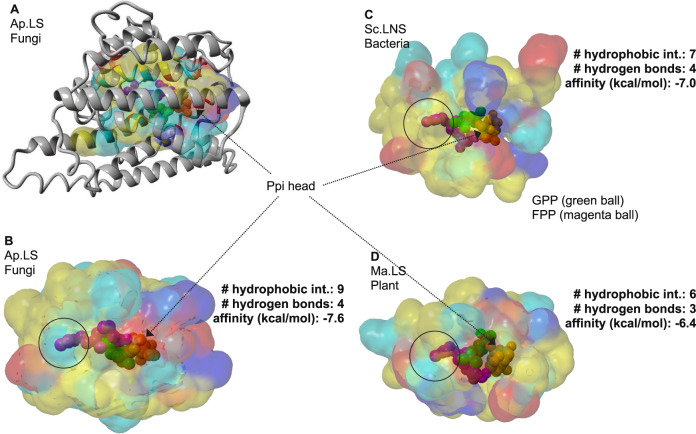
Structural comparison of linalool synthases in bacteria, fungi, and plants. **A** The overlay of 3D structure (gray ribbon) and substrate-binding pocket of Ap.LS. Energy minimized homology model was used with 4LXW as template. GPP and FPP from 2ONG and 6A2C, respectively, were structurally aligned to 4LXW. Binding pocket surfaces for fungal (**B**), bacterial (**C**), and plant (**D**) linalool synthases. GPP (2-fluorogeranyl diphosphate) and FPP ligands are shown as green and magenta spheres, respectively. The solvent-accessible surface of residues in the binding pocket is colored yellow (hydrophobic), cyan (polar), red (negative), or blue (positive). Best docking model from AutoDock Vina was used to submit to the Protein-Ligand Interaction Profiler (PLIP) server (Salentin et al.^32^). Ap.LS, the most stable protein–ligand complex has the most hydrophobic interactions with the hydrophobic tail of GPP.

Subsequently, we compared the protein structures of the linalool synthases from the fungus (*A. pediades*), the bacterium (*S. clavuligerus*), and the plant (*M. aquatica*). The crystal structure of Sc.LNS is 5NX5 (PDB ID)^10^. The homolog models of Ap.LS and Ma.LS (Q8H2B4) were built based on the crystal structure of (+)-bornyl diphosphate synthase from *Salvia officinalis* (1N1B/1N21)^28^ and 1,8-cineole synthase from *S. clavuligerus* (5NX7)^10^, respectively. As the sequence similarity between Ap.LS and 1,8-cineole is only 20.3% (and active-site identity is 49%), the Ap.LS model may have some deviations from its real structure. Nevertheless, their active-site regions are highly conserved (Fig. 6A). Fungal linalool synthase and bacterial linalool synthase are much more similar to each other than to plant linalool synthase in both active-site regions (Supplementary Table S4) and overall structures (Fig. 6B–D). As a typical plant monoterpene synthase, Ma.LS has two domains (α and β domains) and thus is noticeably larger than the other two synthases with active site residing only in the α domain (catalytic domain, Supplementary Fig. S8). In contrast, microbial linalool synthases, Sc.LNS and Ap.LS, are similar to typical class I terpene cyclases with a single domain, despite with acyclic products (Supplementary Fig. S9). Both GPP (its analog, 2-fluorogeranyl diphosphate) and FPP were docked into the three models. We mainly analyzed the interactions of the three linalool synthases with GPP. With 9 hydrophobic interactions with GPP, Ap.LS had the highest amount, as compared to 7 of Sc.LNS and 6 of Ma.LS (Fig. 6B–D). Except for the negatively charged pyrophosphate (Ppi) head, GPP is largely hydrophobic, thus these hydrophobic interactions may contribute to the high activity of Ap.LS. The number of hydrogen bonds identified for the three enzymes was similar. In addition, Ap.LS had the highest binding affinity (−7.6 kcal/mol) to GPP, followed by Sc.LNS (−7 kcal/mol) and Ma.LS (−6.4 kcal/mol). The binding affinity inversely correlated with the *K*_m_ values of the three enzymes (Table 1), where higher binding affinity contributed to a lower *K*_m_ value. As compared for Ap.LS and Ap.LNS, Ap.LS has higher binding affinity to GPP than Ap.LNS (−7.3 kcal/mol) but lower binding affinity (−8.5 kcal/mol) to FPP than Ap.LNS (−9.0 kcal/mol). The binding affinity data are nicely correlated with their difference in monoterpene and sesquiterpene activities.

**Fig. 7:**
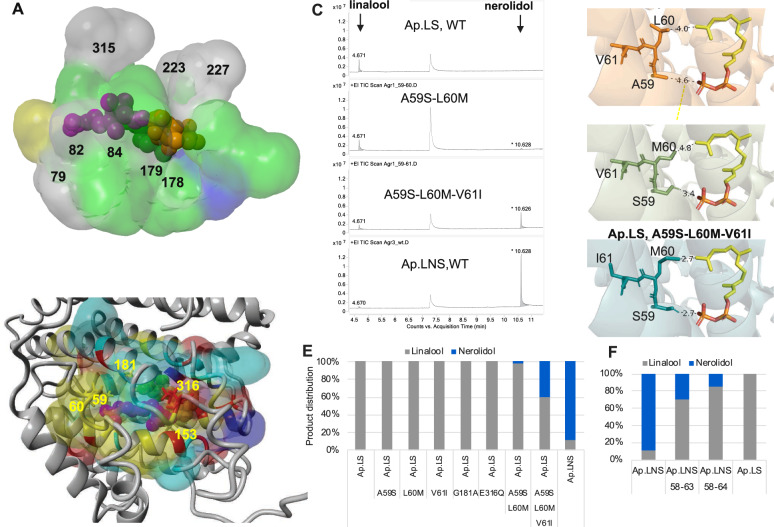
Structure analysis for the understanding of activity and specificity. **A** The aligned 3D active site surfaces of Ap.LS, Sc.LNS, and Ma.LS. The residues are listed in Supplementary Table S4. Gray: conserved residues among the linalool synthases of three kingdoms; green: not conserved in plant linalool synthase; blue: not conserved in bacterial linalool synthase; yellow: not conserved in fungal linalool synthase. **B** Highlight of the key residue difference between Ap.LS and Ap.LNS. Detailed comparison of binding-pocket residues in Supplementary Table S5. **C** Mutation of the key residues responsible for Ap.LS specificity. **D** The interactions of the selected residues with FPP in Ap.LS wild type (WT) and its mutants (distance by dash line, unit: Å). **E** Quantitative comparison of Ap.LS and its mutants. **F** Quantitative comparison of Ap.LNS and its mutants.

Furthermore, we superimposed the 3D structures of the active sites of the three enzymes (Fig. 7A and Supplementary Table S4). Residues in the binding pocket of plant linalool synthase showed the greatest divergence (green regions, Fig. 7A), although structure folding remains conserved. Overall, as shown in the gray regions (Fig. 7A) and highlighted in Supplementary Table S4, there were 8 conserved residues among the three enzymes, including the aspartate-rich motif, D(D/E)XXD, responsible for Mg^2+^ cofactor and substrate binding^1^ and NSE triad, (N/D)Dxx(S/T)xxxE, responsible for the substrate binding and coordination of the diphosphate and trinuclear Mg^2+^ [PPi-(Mg^2+^)_3_] cluster^1^. All these structural analyses partially explain the activity difference among the three linalool synthases from different kingdoms. Nevertheless, there are other factors that might also contribute to the high in vivo activity of Ap.LS in *E. coli*, such as the non-active-site residues, protein expression, and solubility (Supplementary Fig. S6).

Through the structural comparison of linalool synthases from different species, we have observed that the binding affinities of enzymes to the substrates (GPP or FPP) nicely correlated with their activities, especially the *K*_m_ values, where higher binding affinity typically contributes to a lower *K*_m_ value. However, it is much more complex to explain the difference in *k*_cat_ values, due to the large structural difference among these enzymes. To address these, molecular dynamics simulation is advantageous in evaluating the dynamic interactions between enzyme and substrates, intermediates, and products. To do that, it is more appropriate to use the accurate crystal structures of these enzymes, which is one of our future works.

### Mechanism study on the Ap.LS selectivity

Lastly, we attempted to understand the specificity of Ap.LS as compared to other fungal LNSs. Particularly, Ap.LNS and Ap.LS share the highest identity 77.9%; hence, we compared their difference of residues surrounding the substrate-binding pocket. In total, five residues were found to be different (Fig. 7B and Supplementary Table S5): (1) in the Ppi head region (A59:S58, E316:Q315, I153:V152, here, the former and latter residues refer to that of Ap.LS and Ap.LNS, respectively, especially that E316 is expected to have charged interactions with the Ppi group of GPP/FPP); (2) in the FPP tail region (L60:M59, which could affect the interaction with FPP); and (3) in GPP tail region (G181:A180, which might affect flexibility of helix where the tail end of GPP resides). A series of single mutants were constructed for Ap.LS, A59S, L60M, G181A, and E316Q. However, none of these single mutants had effect on the selectivity of Ap.LS (Fig. 7E). We speculated that the specificity might be the synergistic result of multiple residues. The region A59–L60 was particularly interesting, as A59 and L60 are in close proximity to both the Ppi head and hydrocarbon tail of FPP (Fig. 7D). Indeed, the combination of A59S and L60M mutations resulted in the production of a trace amount of nerolidol, ~2% of total amount of linalool and nerolidol produced (Fig. 7C, E), which indicated that the two mutations are sufficient to convert Ap.LS from a monofunctional linalool synthase to a bifunctional LNS. Adjacent to A59–L60, another residue is also different between Ap.LS (V61) and Ap.LNS (I60). Although single mutation V61I had no effect on the selectivity of the wild-type Ap.LS, the introduction of V61I enhanced the nerolidol production by 12-fold (~40% of total linalool and nerolidol produced) and decreased linalool production by 45% on the basis of the double mutant A59S–L60M (Fig. 7C, E). It seems that the mutation L60M–A59S favors sesquiterpene activity (nerolidol formation) by stabilizing the ligand in a favorable position (A59S) and by promoting the easier leave of the Ppi group from the binding pocket (L60M, Fig. 7D). The third mutation V61I further enhances the effect by pushing M60 and S59 closer to FPP (Fig. 7D).

In addition, we also combined the mutations of two regions and obtained the quadruple mutant A59S–L60M–V61I–E316Q; however, it did not further increase nerolidol production. Furthermore, we observed that the wild-type Gm.LNS, whose corresponding residues are more similar to those 59–61 of Ap.LS (S59:A59, L60:L60, V61:V61, Supplementary Fig. S9), produced the highest amount of linalool (15%) among all the wild-type fungal LNSs (Fig. 3C). As such, we concluded that the region residues (A59–V61) play an essential role for the high specificity of Ap.LS.

Lastly, we further tested whether the mutation of the same region could change the selectivity of Ap.LNS to produce only linalool. By introducing 5 mutations (S58A+M59L+I60V+T62P+V63L, or “58–63” mutant in Fig. 7F) or 6 mutations (S58A+M59L+I60V+T62P+V63L+E64G, or “58–64” mutant), linalool percentage was increased from 11% (wild type) to 86% (“58–64” mutant). However, none of the mutation could completely eliminate the nerolidol production, indicating that additional residues are also playing roles in regulating the selectivity.

Here, aided by structural comparison of Ap.LS and Ap.LNS, we managed to identify the key residues that alter the selectivity of Ap.LS. The success here has two broad meanings. First, such a structure-based method is of general application in understanding the catalytic mechanism. Our method works even without protein crystal structures but using homology model. And recent achievement by the Google DeepMind’s AlphaFold 2 (https://deepmind.com/blog/article/alphafold-a-solution-to-a-50-year-old-grand-challenge-in-biology) can further support our method. Second, the identification of key residues inspires future studies, such as the rational design and engineering of linalool synthases, nerolidol synthases, or bifunctional linalool and nerolidol synthases. More broadly, it might also encourage studies in understanding the selectivities of various TPSs. It was known that some are highly specific and have only a single product, whereas others have multiple products, particularly, the γ-humulene synthase from *Abies grandis*, which generates 52 different sesquiterpenes^29^. The underlying catalytic mechanism is fascinating but not fully understood. Our study here provides some insights, and further research is required.

## Conclusion

In this study, we have applied BLAST search, full-sequence alignment, and active-site alignment to search for fungal linalool synthases. The combined use of these bioinformatic tools enabled us to identify three mushroom LNSs and a highly specific and active monofunctional linalool synthase. Such a workflow is of universal value for the rapid identification of other unknown enzymes, not limited to TPSs. The discovery of Ap.LS illustrates the importance and necessity to further explore fungal genomes for other valuable biocatalysts. Phylogenetic analysis indicated that fungal enzymes evolved divergently from plants and bacteria. The in vitro characterization of purified Aa.LNS and Ap.LS provided kinetic parameters and further re-validated that Ap.LS is highly specific. The in vivo study proved that Ap.LS has a higher linalool yield and/or purity as compared to the bacterial Sc.LNS and plant Cb.LS when expressed in *E. coli*. Thus Ap.LS is more suitable for metabolic engineering applications. Furthermore, structural analysis demonstrated that the binding affinities could explain the *K*_m_ value differences of linalool synthases from different kingdoms. Through structural comparison and mutation experiments, we identified the two key residues responsible for the selectivity of Ap.LS. By mutating these residues, we can change Ap.LS from a monofunctional to a bifunctional enzyme. To further understand the catalytic mechanism, the crystal structure of Ap.LS is required, which will be addressed in our future works.

## Methods

### Sequence validation of AAE3_109435 in the genome

AAE3_109435 sequence was analyzed from Illumina sequencing data with manual intron annotation. To verify the presence of the gene AAE3_109435, genomic DNA (gDNA) of *A. aegerita* was first extracted. Briefly, the vegetative mycelia of *A. aegerita* was ground with pestle and mortar in liquid nitrogen. Around 200 mg of ground mycelium was transferred in 1.5 ml E-cups and 500 µl of lysis buffer (400 mM Tris-HCl pH 8.0, 60 mM EDTA pH 8.0, 150 mM NaCl, 1% sodium dodecyl sulfate) was added. The solution was vortexed and incubated at ambient temperature for 10 min before the addition of 150 µl potassium acetate (3 M, pH 4.8). The solution was then vortexed and centrifuged for 10 min at 3000 × *g*. The supernatant containing gDNA was transferred into a new E-cup and the gDNA was obtained by ethanol precipitation method. The gDNA was used as the PCR template to amplify AAE3_109435 with primers AAE3_9435f (CCAAGATTGTCGTCAACGCC) and AAE3_9435r (CTGTGGGCCATTCTGTCCAT). The amplified region was subsequently sequenced and confirmed (results in Supplementary Fig. S1).

### Bioinformatics prediction of fungal linalool synthases

The fungal linalool synthase candidate genes were obtained by the combination of BLAST search in JGI fungal genomics and UniProt databases, full-sequence alignment, and predicted active-site alignment. Those homologs with the highest similarity to Aa.LNS or Ap.LS in full sequences and in active sites were selected as the targets for experimental validation. The high similarity is defined by >50% identity, and enzymes of high similarity are typically in the same cluster of the phylogenetic tree or in the sequence similarity network^3^.

### Prediction of active sites of fungal TPSs

The full-sequence alignment was generated by aligning the complete sequences of linalool synthase and LNS proteins from the three kingdoms by Clustal Omega program v1.2.2. In contrast, active-site alignment was proceeded by identifying amino acid residues surrounding the predicted active sites of each enzyme in 3D structures. We used an in-house developed algorithm, BioTransformer v0.9, to predict and align the active sites. In brief, the algorithm first searches the PDB for appropriate templates. Next, the user gets to select the most appropriate PDB template or templates with the most appropriate ligands (usually the ligands or combination of ligands that maximize the space within the active site). Using this approach, the PDB structures 4LXW (Epi-isozizaene synthase in complex with inorganic pyrophosphate (Ppi) and benzyl triethyl ammonium from *S. coelicolor*) and 5NX5 (LNS in complex with 2-fluorogeranyl diphosphate from *S. clavuligerus*) were chosen as templates for the prediction of the active sites. To maximize the number of residues found within the binding pockets of the PDB structures, residues found within 6.0 Å from the substrate were considered as part of the active site, and the union set derived from both structural templates was used as the predicted active site.

### Phylogenetic analysis and sequence similarity network of TPSs

Full amino acid sequences were used to build a phylogenetic tree. Alignment was carried out by Clustal Omega program version 1.2.2 and the phylogenetic analysis was conducted with the Neighbor-Joining method or Maximum Likelihood method by MEGA version 7.0.26. For the predicted active sites of TPSs, the Enzyme Function Initiative-Enzyme Similarity Tool (EFI-EST, http://efi.igb.illinois.edu/efi-est/) was used to generate sequence similarity networks (SSNs) with the database-independent alignment score of 16. The resulting SSNs were visualized using the open-source software Cytoscape version 3.5.1.

### Structural modeling and analysis

The homolog models of Ap.LS and Ma.LS (Q8H2B4) from *M. aquatica* were built based on the crystal structure of the most closely related TPSs: Epi-isozizaene synthase from *S. coelicolor* (PDB ID, 4LXW), 1,8-cineole synthase from *S. clavuligerus* (PDB ID, 5NX7)^10^, and (+)-bornyl diphosphate synthase from *S. officinalis* (PDB ID, 1N1B/1N21)^28^, using the Modeller software (https://salilab.org/modeller/). The binding pockets, consisting of 21–22 residues within 6 Å from the substrate, were determined by the PyMOL software v2.1.1. Structural alignment of proteins was conducted using MUSTANG^30^ implemented in YASARA^31^. The web implementation of PLIP (Protein-Ligand Interaction Profiler) was used to identify interactions between ligand and the surrounding amino acid residues in the protein^32^. Docking was performed with AutoDock Vina with an exhaustiveness of 200. AutoDock Tools was used to prepare the PDBQT files for the homology models for the enzymes Ap.LS, Ap.LNS, Sc.LNS, and Ma.LS and also for the ligands GPP and FPP^33^. A search space of 24 × 24 × 24 Å was used for GPP and a search space of 24 × 28 × 24 Å was used for FPP.

### Plasmid and strain construction

The fungal TPSs, Sc.LNS from *S. clavuligerus* and Cb.LS from *C. breweri*, were codon optimized and cloned into the pET-11a vector (Novagen) either alone or together with ispA_S80F (GPP synthase) or ispA (FPP synthase) from *E. coli*. For fungal TPS characterization, the chassis *E. coli* strain was used. The strain carried a plasmid p15A-cam-T7-dxs-idi that overexpresses the enzymes DXS and IDI from *E. coli* to enhance the supply of terpene precursors^3^. For the monoterpene production study, the mevalonate pathway was overexpressed in p15A vectors under the T7 promoter variants^9^.

### Mutation study of Ap.LS and Ap.LNS

The targeted mutations were introduced to Ap.LS and Ap.LNS using in-house methods as described previously^2^. Single mutants (A59S, L60M, V61I, G181A, and E316Q) and the double mutant (A59S–L60M) were directly introduced to the wild type. Triple mutants (A59S–L60M–V61I, A59S–L60M–E316Q) were introduced on top of the double mutant A59S–L60M. E316Q was introduced to the triple mutant A59S–L60M–V61I to obtain the quadruple mutant A59S–L60M–V61I–E316Q. Primers used are as listed in Supplementary Table S6.

### Terpenoid production in *E. coli*

*E. coli Bl21-Gold DE3* strain (Stratagene) was used for linalool production. The strains carrying genes of the mevalonate pathway and linalool synthases from different species were grown in 1 ml of ZYM medium or TB supplemented with 0.4% glucose. The cultures were incubated for 3 days (28 °C, 250 rpm)^2^. In addition, 200 μl of isopropyl myristate was used for harvesting the linalool and/or nerolidol. Antibiotics (34 μg/ml chloramphenicol, 50 μg/ml spectinomycin, and 100 μg/ml ampicillin) were supplemented to maintain the plasmids. ZYM medium was prepared as previously described^2^ (1% tryptone, 0.5% yeast extract, 25 mM Na_2_HPO_4_, 25 mM KH_2_PO_4_, 50 mM NH_4_Cl, 5 mM Na_2_SO_4_, 2 mM MgSO_4_, 0.5% glycerol, 0.05% glucose, 15 mM of α-lactose). In ZYM medium, the *E. coli* strains were automatically induced with the depletion of glucose and initiation of lactose consumption.

### Expression and purification of linalool synthases

*E. coli Bl21-Gold DE3* strains (Stratagene) carrying pET-11a-Aa.LNS or pET-11a-Ap.LS and the chaperone plasmid pTf16 (Takara Bio Inc., Japan) were grown in 4 l of 2× ZYM media with antibiotics (100 μg/ml ampicillin and 34 μg/ml chloramphenicol) at 20 °C and 225 rpm. The cells were induced with 0.1 mM IPTG (to express linalool synthase/LNSs) and 3 mM of arabinose (to express chaperone proteins) when OD600 reached 0.6–0.8. The cells were further cultured for another 20 h and harvested. Cells were washed and re-suspended in 100 ml of the solution (50 mM Tris of pH 8, 500 mM NaCl, 0.1% TritonX100, 5% glycerol) with 2 tablets of protease cocktail inhibitor (Sigma-Aldrich, Singapore). The cells in the suspension were lysed by sonication at 4 °C (5 s ON and 5 s OFF, 40% amplitude). The proteins in the supernatant were then extracted by 1 ml × HisTrap column. The column with bound proteins was washed by His-binding buffer (50 mM Tris of pH 8, 500 mM NaCl, and 20 mM imidazole) and eluted by 20 ml of His-elution buffer (50 mM Tris of pH 8, 500 mM NaCl, and 500 mM imidazole). The eluted protein was further concentrated by Vivaspin® 10 kDa cutoff spin column (Merck, Singapore). In the end, 2.7 mg/ml of Aa.LNS (16.3% purity) and 22.2 mg/ml of Ap.LS (71.2% purity) were obtained.

### Kinetic analysis

Steady-state kinetics of purified Aa.LNS and Ap.LS were determined by measuring PPi release via conversion to phosphate with inorganic pyrophosphatase in the EnzChek® Pyrophosphate Assay Kit (Thermo Fisher Scientific, Singapore). Substrate concentrations of GPP and (E,E)-FPP (Echelon bioscience, USA) were varied between 6.5 and 40 μM. Reactions were carried with 20 μg/ml (460 nM) of Aa.LNS or 10 μg/ml (250 nM) of Ap.LS at 37 °C for 1 h. PPi concentrations were calculated by linear interpolation of the standard curve using the kit assay (0–60 μM). The scatter plots of initial rate versus substrate concentration were fitted to the equation *v* = *v*_max_[*S*]/(*K*_*M*_ + [*S*]), where *v*_max_ = *k*_cat_ [*E*_0_].

### Gas chromatography–mass spectrometry (GC-MS) detection and quantification of terpenes

For characterization of fungal TPSs, the headspace compounds were sampled at 60 °C for 20 min by SPME with a DVB/CAR/PDMS (50/30 µm divinylbenzene/carboxen/polydimethylsiloxane) fiber (length 1 cm; Supelco, Steinheim, Germany). Subsequently, the compounds were desorbed for 1 min in the split inlet (250 °C; SPME liner, 0.75 mm i.d.; Supelco) and analyzed by an Agilent 7980B GC equipped with an Agilent 5977B MSD. Samples were injected into Agilent DB5ms column with a split ratio of 40:1 at 240 °C. The oven program started at 80 °C for 1 min, was raised up to 210 °C at 10 °C/min, then to 310 °C at 60 °C/min and maintained at 310 °C for another 2 min. Mass spectrometer was operated in EI mode with full scan analysis (*m*/*z* 33–300, 9 scans/s). In addition to mass spectra, Kovats retention indices of the detected compounds were calculated by calibrating with a C8–C30 alkane mix and compared with literature data in the National Institute of Standards and Technology database (Supplementary Fig. S2).

For quantification of linalool and nerolidol of different strains, the organic layer with secreted terpenes was separated and diluted with ethyl acetate by 10–100 times. The samples were then analyzed with the same GC-MS program as the characterization method. The concentrations were calculated by interpolation using the standard curve of authentic linalool and nerolidol standards (Sigma-Aldrich, Singapore).

### Chiral study of linalool produced by fungal linalool synthases

The chirality of linalool produced by fungal linalool synthases was analyzed by the GC chiral CycloSil-B column (30 m, 0.25 mm, 0.25 µm, Agilent, Singapore) in the same Agilent 7980B GC equipped with the 5977B MSD. The oven program started at 80 °C for 2 min, was raised up to 210 °C at 5 °C/min, then to 250 °C at 20 °C/min and maintained at 250 °C for another 2 min. Mass spectrometer was operated in EI mode with full scan analysis (*m*/*z* 33–300, 5.5 scans/s). The retention times and mass spectra of samples were compared with authentic standards of both (*R*)-linalool and a mixture of (*R*/*S*)-linalool.

### Statistics and reproducibility

General data analysis (means and standard deviation) was performed primarily by Python V.3.8.3. For the production of linalool and/or nerolidol, three biological replicates (different colonies) were used for each condition.

### Reporting summary

Further information on research design is available in the Nature Research Reporting Summary linked to this article.

## Supplementary information

Supplementary Information

Description of Additional Supplementary Files

Supplementary Data 1

Supplementary Data 2

Supplementary Data 3

Reporting Summary

## Data Availability

All data needed to evaluate the conclusions in the paper are present in the paper and/or the Supplementary Materials. Additional data related to this paper may be requested from the authors.
