# Supplementary material for: Bioinformatics-aided identification, characterization and applications of mushroom linalool synthases
Source: Commun Biol. 2021 Feb 17;4:223. doi: 10.1038/s42003-021-01715-z (PMC7890063; doi:10.1038/s42003-021-01715-z)
Supplement: Supplementary file 1 — Supplementary Information [file 42003_2021_1715_MOESM1_ESM.pdf]

Supporting information

Bioinformatics-Aided Identification, Characterization and Applications of Mushroom Linalool Synthases

Congqiang Zhang[1*], Xixian Chen[1], Raphael Tze Chuen Lee[2], Rehka T[1], Sebastian Maurer-Stroh[2,3#], Martin Rühl[4#]

1. Singapore Institute of Food and Biotechnology Innovation (SIFBI), Agency for Science, Technology and Research (A*STAR), Singapore

2. Bioinformatics Institute (BII), Agency for Science Technology and Research (A*STAR), 30 Biopolis Street, #07-01 Matrix, 138671, Singapore

3. Department of Biological Sciences (DBS), National University of Singapore (NUS), Singapore

4. Institute of Food Chemistry and Food Biotechnology, Justus Liebig University Giessen, Giessen, Germany

[#]equal contribution

*To whom correspondence may be addressed. Email:

zcqsimon@outlook.com or congqiang_zhang@sifbi.a-star.edu.sg

Table of contents

Table S1. Predicted active sites of terpene synthases with 4LXW and 5NX5 as the templates.
Table S2. Comparison and combination of the predicted active sites of the two templates 4LXW and 5NX5.
Table S3. Summary of LSs and LNSs in this study.
Table S4. 3D alignment of active-site residues of Ap.LS, Sc.LNS and Ma.LS.
Table S5. Comparison of residues in the substrate-binding pockets of Ap.LS and Ap.LNS.
Table S6. Primers used for Ap.LS mutation.
Fig. S1. Nucleic acid sequence of AAE3_109435 in the genome.
Fig. S2. Mass spectra and retention indices for terpenes detected in this study.
Fig. S3. The alignment between Agrped_689675 and Galma_223690 and BLAST search results in UniProt database with Agrped_689675 (or Ap.LS).
Fig. S4. GCMS chromatograms and spectra for Galma_266794.
Fig. S5. Amino acid sequence alignment and identity table of LSs and LNSs from fungi, bacteria and plants.
Fig. S6. Expression and solubility analysis of the fungal LNSs and LS.
Fig. S7. The optimization of Aa.LNS solubility with the co-expression of chaperone proteins.
Fig. S8. The sequence alignment and their secondary structures of plant terpene synthases.
Fig. S9. The sequence alignment and their secondary structures of microbial terpene synthases.
Fig. S10. Purification of Aa.LNS, full image for Fig. 4A.
Fig. S11. Purification of Ap.LS, full image for Fig. 4B.

Table S1. Predicted active sites of terpene synthases with 4LXW and 5NX5 as the templates. Residues that are within 6.0 Angstrom from the ligands in these structures were considered as being part of the enzyme active site. The predicted active sites listed below is derived from the union set from both structural templates.

No.	Homologues	Predicted active sites by BioTransfomer v0.9 or PyMOL v2.1.1																																			
0	**consensus**	**X**	**L**	**X**	**M**	**X**	**F**	**V**	**D**	**E**	**T**	**D**	**V**	**E**	**Y**	**R**	**T**	**X**	**G**	**X**	**X**	**X**	**X**	**N**	**D**	**S**	**Y**	**X**	**E**	**X**	**X**	**H**	**N**	**W**	**X**	**W**	**S**	**R**	**Y**
1	5NX5[1]	V	I	G	L	T	F	L	D	D	F	D	W	K	H	R	T	I	C	C	L	V	I	N	E	S	F	K	D	-	Q	H	N	F	Y	W	G	R	Y
2	4LXW[1]	Y	V	M	S	F	F	V	D	D	H	D	Y	E	Y	R	T	F	A	H	W	A	A	N	D	S	L	K	E	-	E	H	N	W	V	F	H	R	Y
3	D5SL78	V	I	G	L	T	F	L	D	D	F	D	W	K	H	R	T	I	C	C	L	V	I	N	E	S	F	K	D	-	Q	H	N	F	Y	W	G	R	Y
4	D8RNZ9	I	Y	A	L	V	F	L	D	D	L	E	V	Q	Y	R	T	G	F	M	V	V	I	N	D	S	F	K	E	G	Y	H	N	V	S	W	S	R	Y
5	Agr6	F	L	A	M	F	F	V	D	E	T	D	V	Q	Y	R	T	I	G	A	S	L	I	N	D	S	Y	V	E	R	D	H	N	W	N	W	S	R	Y
6	Agr2	F	I	T	H	L	W	F	D	E	T	D	G	E	Y	R	T	S	G	A	C	I	F	N	D	S	Y	M	E	K	N	A	N	W	N	W	S	R	Y
7	Agr3	S	L	T	L	L	F	H	D	N	S	D	V	Q	Y	R	T	S	G	C	S	V	T	N	D	S	Y	V	E	R	D	H	N	W	S	W	S	R	Y
8	Agr5	F	S	N	T	L	W	L	D	E	T	D	V	E	Y	R	T	S	A	V	C	V	F	N	D	S	Y	M	E	K	S	A	N	W	N	W	S	R	Y
9	Agrped1_109003	A	L	T	M	L	F	H	D	N	S	D	V	Q	Y	R	T	S	G	C	C	V	T	N	D	S	Y	V	E	R	D	H	N	W	S	W	S	R	Y
10	Agrped1_640059	C	L	T	L	L	F	H	D	N	S	D	V	Q	Y	R	T	S	G	C	C	V	T	N	D	S	Y	V	E	K	D	H	N	W	S	W	S	R	Y
11	Agrped1_665597	F	A	T	M	F	F	V	D	E	T	D	C	Q	Y	R	N	V	G	A	T	V	I	N	D	S	Y	R	E	T	D	H	N	W	N	W	S	R	Y
12	Agrped1_693394	F	L	A	M	F	F	V	D	E	T	D	C	Q	Y	R	N	I	G	A	S	I	I	N	D	S	Y	K	E	T	D	H	N	W	N	W	N	R	Y
**13**	**Agrped1_694262**	**F**	**L**	**A**	**M**	**Y**	**F**	**V**	**D**	**E**	**T**	**D**	**V**	**E**	**Y**	**R**	**T**	**S**	**A**	**G**	**T**	**I**	**I**	**N**	**D**	**S**	**F**	**R**	**E**	**C**	**G**	**H**	**N**	**W**	**N**	**W**	**S**	**R**	**Y**
14	Agrped1_749682	N	L	C	V	L	F	V	D	E	S	D	V	E	F	R	N	S	A	V	C	V	C	N	D	S	Y	M	E	K	M	N	N	W	N	W	S	R	Y
15	Agrped1_804996	-	-	F	M	F	F	V	D	E	T	D	C	Q	Y	R	N	V	G	I	S	I	I	N	D	S	Y	R	E	T	D	H	N	W	N	W	N	R	Y
**16**	**Agr8**	**-**	**L**	**A**	**M**	**Y**	**F**	**V**	**D**	**E**	**T**	**D**	**V**	**E**	**Y**	**R**	**T**	**C**	**G**	**A**	**T**	**I**	**I**	**N**	**D**	**S**	**Y**	**R**	**E**	**C**	**E**	**H**	**N**	**W**	**N**	**W**	**S**	**R**	**Y**
**17**	**Agrped1_820868**	**L**	**L**	**A**	**M**	**Y**	**F**	**I**	**D**	**E**	**T**	**D**	**V**	**E**	**Y**	**R**	**T**	**C**	**G**	**A**	**T**	**I**	**I**	**N**	**D**	**S**	**Y**	**R**	**E**	**C**	**G**	**H**	**N**	**W**	**N**	**W**	**S**	**R**	**Y**
**18**	**M413_27416**	**L**	**L**	**A**	**M**	**Y**	**F**	**V**	**D**	**E**	**T**	**D**	**V**	**E**	**Y**	**R**	**T**	**C**	**G**	**A**	**T**	**I**	**I**	**N**	**D**	**S**	**Y**	**R**	**E**	**C**	**A**	**H**	**N**	**W**	**N**	**W**	**S**	**R**	**Y**
**19**	**Galma_266794**	**L**	**L**	**A**	**M**	**Y**	**F**	**V**	**D**	**E**	**T**	**D**	**V**	**E**	**Y**	**R**	**T**	**C**	**G**	**A**	**T**	**I**	**I**	**N**	**D**	**S**	**Y**	**R**	**E**	**C**	**A**	**H**	**N**	**W**	**N**	**W**	**S**	**R**	**Y**
**20**	**Galma_63556**	**-**	**-**	**F**	**M**	**Y**	**F**	**A**	**D**	**E**	**T**	**D**	**I**	**E**	**Y**	**R**	**T**	**C**	**G**	**A**	**S**	**I**	**A**	**N**	**D**	**S**	**Y**	**L**	**E**	**R**	**D**	**H**	**N**	**C**	**Y**	**W**	**S**	**R**	**Y**
**21**	**Hypsu1_148365**	**-**	**-**	**F**	**M**	**Y**	**F**	**A**	**D**	**E**	**T**	**D**	**V**	**E**	**Y**	**R**	**T**	**C**	**G**	**G**	**S**	**I**	**A**	**N**	**D**	**S**	**Y**	**L**	**E**	**R**	**D**	**H**	**N**	**C**	**Y**	**W**	**S**	**R**	**Y**
**22**	**Hypsu1_148385**	**F**	**L**	**A**	**M**	**Y**	**F**	**A**	**D**	**E**	**T**	**D**	**V**	**E**	**Y**	**R**	**T**	**C**	**G**	**A**	**S**	**I**	**A**	**N**	**D**	**S**	**Y**	**L**	**E**	**R**	**D**	**H**	**N**	**C**	**Y**	**W**	**S**	**R**	**Y**
**23**	**Agrped1_689671**	**F**	**L**	**G**	**M**	**Y**	**F**	**A**	**D**	**E**	**T**	**D**	**V**	**E**	**Y**	**R**	**T**	**C**	**G**	**A**	**S**	**I**	**A**	**N**	**D**	**S**	**Y**	**L**	**E**	**R**	**D**	**H**	**N**	**C**	**Y**	**W**	**S**	**R**	**Y**
**24**	**Agrped1_689675**	**F**	**L**	**G**	**M**	**Y**	**F**	**A**	**D**	**E**	**T**	**D**	**I**	**E**	**Y**	**R**	**T**	**C**	**G**	**G**	**S**	**I**	**A**	**N**	**D**	**S**	**Y**	**L**	**E**	**R**	**D**	**H**	**N**	**C**	**Y**	**W**	**S**	**R**	**Y**
**25**	**Galma_223690**	**F**	**L**	**G**	**M**	**Y**	**F**	**A**	**D**	**E**	**T**	**D**	**V**	**E**	**Y**	**R**	**T**	**C**	**G**	**A**	**S**	**I**	**A**	**N**	**D**	**S**	**Y**	**L**	**E**	**R**	**D**	**H**	**N**	**C**	**Y**	**W**	**S**	**R**	**Y**
26	Agrped1_804989	-	-	-	M	F	L	V	D	K	T	D	C	Q	Y	R	N	V	G	A	A	I	T	N	D	S	Y	K	E	T	D	H	N	W	N	W	N	R	Y
**27**	**Agr11**	**T**	**L**	**A**	**M**	**F**	**F**	**V**	**D**	**E**	**T**	**D**	**V**	**E**	**Y**	**R**	**T**	**S**	**G**	**A**	**M**	**T**	**-**	**-**	**-**	**-**	**-**	**-**	**R**	**S**	**A**	**H**	**N**	**A**	**E**	**W**	**A**	**R**	**Y**
28	Agr1	C	L	T	L	L	F	H	D	N	S	D	V	Q	Y	R	T	S	G	C	C	V	T	N	D	S	Y	V	E	K	H	H	N	W	S	W	S	R	Y
29	Agr9	F	L	A	M	F	Y	V	D	E	T	D	V	E	Y	R	S	S	G	C	S	I	A	N	D	S	Y	M	E	R	E	H	N	W	N	W	T	R	Y
30	Agr4	N	L	C	V	L	F	V	D	E	S	D	V	E	F	R	N	S	A	V	C	V	C	N	D	S	Y	M	E	K	S	N	N	W	N	W	S	R	Y
31	Agr7	F	L	A	M	F	F	V	D	E	S	D	V	Q	Y	R	T	I	G	A	S	L	C	N	D	S	Y	V	E	R	D	H	N	W	N	W	S	R	Y
32	AAE3_109435	T	L	A	M	F	F	V	D	E	T	D	V	E	Y	R	T	S	G	A	M	Y	C	N	D	S	Y	S	E	S	A	H	N	A	E	W	A	R	Y
**33**	**Agr10**	**T**	**L**	**A**	**M**	**F**	**Y**	**V**	**D**	**E**	**A**	**D**	**W**	**K**	**-**	**-**	**-**	**-**	**-**	**-**	**T**	**I**	**G**	**N**	**D**	**S**	**Y**	**M**	**K**	**-**	**-**	**-**	**R**	**W**	**N**	**W**	**T**	**R**	**Y**
34	Agrped1_705454	P	G	H	Y	I	V	M	D	D	T	G	L	Q	F	Y	T	T	K	G	G	L	T	N	P	G	F	Y	E	R	N	A	E	W	K	W	A	M	Y
35	Q8H2B4[2]	-	-	R	W	I	I	T	D	D	Y	D	Y	E	Y	A	T	I	G	A	V	V	R	D	D	F	R	K	L	-	Y	-	-	-	-	-	-	-	-

Murolene/cadinene synthase cluster and LS/LNS cluster predicted or characterized in this study were shown in blue and green, respectively (consistent with Fig. 3). The non-functional synthase Agr10 and Agr11 were highlighted in red.

[1] The two proteins were used as templates: D5SL78, Sc.LNS from *Streptomyces clavuligerus,* (PDB ID: 5NX5) and Q9K499, Epi-isozizaene synthase from *Streptomyces coelicolor* (PDB ID: 4LXW). For 4LXW, the ligands BTM, POP and MG were used for prediction of active sites. For 5NX5, the ligands 0FV and MG were used for prediction of active sites.

[2] Analyzed by PyMOL software version 2.1.1. The homologue model of Q8H2B4 was built on the structure of (+)-bornyl diphosphate synthase from *Salvia officinalis* (PDB entry ID, 1n1b/1n21) with Modeller software.

Table S2. Comparison and combination of the predicted active sites of the two templates 4LXW and 5NX5. Residues that are within 6.0 Angstrom from the ligands BTM, POP, 0FV and MG found in these structures were considered as being part of the enzyme active site. Since BTM and POP from 4LXW occupies a larger surface area than 0FV from 5NX5, 22 out of 25 active site residues found from the 5NX5 template overlaps with those found from the 4LXW template.

**Index Position**	**52**	**55**	**56**	**76**	**79**	**80**	**81**	**83**	**84**	**86**	**87**	**153**	**156**	**171**	**175**	**178**	**179**	**180**	**181**	**184**	**217**	**218**	**221**	**222**	**225**	**226**	**228**	**229**	**232**	**235**	**237**	**238**	**299**	**303**	**306**	**307**	**312**
4LXW_position		72	73	92	95	96		99	100	102	103	172	175	190	194	197	198	199		203	236	237	240	241	244	245	247	248	253	255	256	325	329	332	333	338	339
4LXW_residue		V	M	S	F	F		D	D	H	D	Y	E	Y	R	T	F	A		W	A	A	N	D	S	L	K	E	E	H	N	W	V	F	H	R	Y
5nx5_position	49	52		72	75	76	77	79	80		83				172	175	176	177	178	181	214		218		222		225	226	229	231			295	299	302		
5nx5_residue	V	I		L	T	F	L	D	D		D				R	T	I	C	C	L	V		N		S		K	D	Q	H			F	Y	W		
5NX5_m[1]	V	I	G	L	T	F	L	D	D	F	D	W	K	H	R	T	I	C	C	L	V	I	N	E	S	F	K	D	Q	H	N	F	Y	W	G	R	Y
4LXW_m[1]	Y	V	M	S	F	F	V	D	D	H	D	Y	E	Y	R	T	F	A	H	W	A	A	N	D	S	L	K	E	E	H	N	W	V	F	H	R	Y

[1] Individual active sites of 4LXM and 5NX5 were predicted with BioTransformer v0.9, which are identical to PyMOL software prediction. Furthermore, to cover more broadly in our model, the active sites of the two templates were merged with each other to obtain 4LXW_m and 5NX5_m, respectively.

Table S3. Summary of LSs and LNSs in this study.

No.	Accession no.	Entry/Gene name	Protein function	Organism	Length
1	Q84ZW8	ACSS_MAIZE	Nerolidol synthase	*Zea mays* (Maize)	590
2	P0CV94	NES1_FRAAN	Nerolidol synthase	*Fragaria ananassa* (Strawberry) (*Fragaria chiloensis x Fragaria virginiana*)	519
3	P0CV95	NES2_FRAAN	Nerolidol synthase	*Fragaria ananassa* (Strawberry) (*Fragaria chiloensis x Fragaria virginiana*)	578
4	P0CV96	NES1_FRAVE	Nerolidol synthase	*Fragaria vesca* (Woodland strawberry) (Potentilla vesca)	580
5	Q9SPN0	LLOS1_ARTAN	R-linalool synthase	*Artemisia annua* (Sweet wormwood)	567
6	Q84UV0	LINS_ARATH	S-linalool synthase	*Arabidopsis thaliana* (Mouse-ear cress)	569
7	Q6ZH94	LINS_ORYSJ	S-linalool synthase	*Oryza sativa subsp. japonica* (Rice)	595
8	Q8H2B4	LLOS_MENAQ	R-linalool synthase	*Mentha aquatica* (Water mint)	606
9	Q9SPN1	LLOS5_ARTAN	R-linalool synthase	*Artemisia annua* (Sweet wormwood)	583
10	Q5SBP3	LLOS_OCIBA	R-linalool synthase	*Ocimum basilicum* (Sweet basil)	574
11	Q96376	LIS_CLABR	S-linalool synthase	*Clarkia breweri* (Fairy fans) (Eucharidium breweri)	870
12	Q2XSC5	LALIN_LAVAN	R-linalool synthase	*Lavandula angustifolia* (Lavender)	564
13	R4I6S7	R4I6S7_9MAGN	S-linalool synthase	*Cinnamomum osmophloeum*	585
14	Q29VN2	TPS2_MAIZE	Nerolidol linalool synthase	*Zea mays* (Maize)	581
15	H6WBC5	H6WBC5_VITVI	Nerolidol linalool synthase	*Vitis vinifera* (Grape)	577
16	A0A068B0N9	A0A068B0N9_9ROSA	Nerolidol linalool synthase	*Prunus cerasoides* var. campanulata	618
17	A0A068B6B6	A0A068B6B6_9ROSA	Nerolidol linalool synthase	*Prunus cerasoides* var. campanulata	561
18	A0A072UZ75	A0A072UZ75_MEDTR	Nerolidol linalool synthase	*Medicago truncatula* (Barrel medic) (Medicago tribuloides)	570
19	B1NA84	B1NA84_ANTMA	Nerolidol linalool synthase	*Antirrhinum majus* (Garden snapdragon)	596
20	B1NA83	B1NA83_ANTMA	Nerolidol linalool synthase	*Antirrhinum majus* (Garden snapdragon)	566
21	Q5UB06	Q5UB06_MEDTR	Nerolidol linalool synthase	*Medicago truncatula* (Barrel medic) (Medicago tribuloides)	573
22	G5CV39	G5CV39_SOLLC	Nerolidol linalool synthase	*Solanum lycopersicum* (Tomato) (Lycopersicon esculentum)	563
23	G7KNU1	G7KNU1_MEDTR	Nerolidol linalool synthase	*Medicago truncatula* (Barrel medic) (Medicago tribuloides)	519
24	E5GAH7	E5GAH7_VITVI	Nerolidol linalool synthase	*Vitis vinifera* (Grape)	545
25	A0A1N7T9S5	A0A1N7T9S5_GOSHI	Nerolidol linalool synthase	*Gossypium hirsutum* (Upland cotton) (Gossypium mexicanum)	586
26	A0A061GH69	A0A061GH69_THECC	Nerolidol linalool synthase	*Theobroma cacao* (Cacao) (Cocoa)	583
27	E5GAH4	E5GAH4_VITVI	Nerolidol linalool synthase	*Vitis vinifera* (Grape)	545
28	G7INZ1	G7INZ1_MEDTR	Nerolidol linalool synthase	*Medicago truncatula* (Barrel medic) (Medicago tribuloides)	806
29	E5GAH1	E5GAH1_VITVI	Nerolidol linalool synthase	*Vitis vinifera* (Grape)	584
30	E5GAI1	E5GAI1_VITVI	Nerolidol linalool synthase	*Vitis vinifera* (Grape)	820
31	G7INZ4	G7INZ4_MEDTR	Nerolidol linalool synthase	*Medicago truncatula* (Barrel medic) (Medicago tribuloides)	819
32	E5GAI0	E5GAI0_VITVI	Nerolidol linalool synthase	*Vitis vinifera* (Grape)	840
33	A0A061GEP1	A0A061GEP1_THECC	Nerolidol linalool synthase	*Theobroma cacao* (Cacao) (Cocoa)	809
34	E5GAH3	E5GAH3_VITVI	Nerolidol linalool synthase	*Vitis vinifera* (Grape)	577
35	D8RNZ9	MTS22_SELML	Nerolidol linalool synthase	*Selaginella moellendorffii* (Spikemoss)	368
36	D5SL78	SCLAV_p1185	Nerolidol linalool synthase	*Streptomyces clavuligerus*	333
37	MN146034	AAE3_05024	Not functional	*Agrocybe aegerita* (*Cyclocybe aegerita*)	355
38	MN954676	AAE3_109435	Nerolidol/linalool synthase	*Agrocybe aegerita* (*Cyclocybe aegerita*)	384
39	JGI ID: Agrped1_689671	Agrped1_689671	Nerolidol/linalool synthase	*Agrocybe pediades*	343
40	JGI ID: Agrped1_689675	Agrped1_689675	R-linalool synthase	*Agrocybe pediades*	344
41	A0A067THX9	Galma_223690	Nerolidol/linalool synthase	*Galerina marginata*	343
42	A0A067T8I8	Galma_63556	Possible nerolidol/linalool synthase	*Galerina marginata*	330
43	A0A0D2NA50	Hypsu1_148365	Possible nerolidol/linalool synthase	*Hypholoma sublateritium*	337
44	A0A0D2NH86	Hypsu1_148385	Nerolidol/linalool synthase	*Hypholoma sublateritium*	344
45	A0A348B793	PpSTS25	Myrcene/linalool synthase	*Postia placenta*	332

Table S4. 3D alignment of active-site residues of Ap.LS, Sc.LNS and Ma.LS. Conversed residues among the three enzymes are highlighted in green.

Yellow		Blue		Green	
Ap.LS_pos	Ap.LS_res	Sc.LNS_pos	Sc.LNS_res	Ma.LS_pos	Ma.LS_res
54	F				
56	L	49	V		
77	M	72	L	360	T
78	N	73	G	361	A
79	F	74	W	362	L
80	Y	75	T	363	D
81	F	76	F	364	D
82	A	77	L	365	V
83	F	78	F	366	Y
84	D	79	D	367	D
85	E	80	D	368	I
		82	F		
88	D	83	D		
149	Y				
153	I	150	W	438	Y
156	E	153	K	441	E
160	R	157	R	445	Y
171	Y				
175	R	172	R	460	A
178	T	175	T	463	T
179	C	176	I	464	I
		215	I	504	R
219	N	218	N	507	D
223	S	222	S	511	T
227	E	226	D	515	E
230	R			518	R
235	H				
309	Y	309	Y	579	A
315	L	316	G	586	G

Table S5. Comparison of residues in the substrate-binding pockets of Ap.LS and Ap.LNS. The different residues of the same position are highlighted in green.

LS_Ap_pos	LS_Ap_res	LNS_Ap_pos	LNS_Ap_res
54	F	53	F
56	L	55	L
57	L	56	L
58	G	57	G
59	A	58	S
60	L	59	M
73	S	72	S
74	C	73	C
75	D	74	D
76	L	75	L
77	M	76	M
78	N	77	N
79	F	78	F
80	Y	79	Y
81	F	80	F
82	A	81	A
83	F	82	F
84	D	83	D
85	E	84	E
87	T	86	T
88	D	87	D
149	Y	148	Y
153	I	152	V
156	E	155	E
157	A	156	A
160	R	159	R
171	Y	170	Y
175	R	174	R
178	T	177	T
179	C	178	C
180	G	179	G
181	G	180	A
184	S	183	S

LS_Ap_pos	LS_Ap_res	LNS_Ap_pos	LNS_Ap_res
215	I	214	I
216	A	215	A
219	N	218	N
220	D	219	D
223	S	222	S
224	Y	223	Y
226	L	225	L
227	E	226	E
230	R	229	R
233	D	232	D
235	H	234	H
236	N	235	N
295	C	294	C
299	Y	298	Y
302	W	301	W
304	Y	303	Y
305	E	304	E
306	T	305	T
309	Y	308	Y
310	Y	309	Y
313	N	312	N
315	L	314	L
316	E	315	Q
332	Y	331	Y

Table S6. Primers used for Ap.LS mutation. The mutational regions are highlighted in red and caps.

**Mutations**	**Forward primers**	**Reverse primers**
A59S	tgctgggtTCGctggttggtccgctgggtac	ccagCGAacccagcaggttgaaatcacatgc
L60M	tgggtgcaATGgttggtccgctgggtaccaa	caacCATtgcacccagcaggttgaaatcaca
V61I	tgcactgATTggtccgctgggtaccaaggaa	ggaccAATcagtgcacccagcaggttgaaat
G181A	ctgtggtgCgaaaccgtcgttctcgttctt	ggtttcGcaccacaggtgtcacggc
E316Q	aatggcctgCaaatccagaaaactcgtcaga	tctggatttGcaggccatttttaccgtaata
A59S-L60M	gggtTcaAtggttggtccgctgggtac	gaccaaccaTtgAacccagcaggttgaa
A59S-L60M-V61I	ggtTcaAtgAttggtccgctgggtacca	cggaccaaTcaTtgAacccagcaggttga

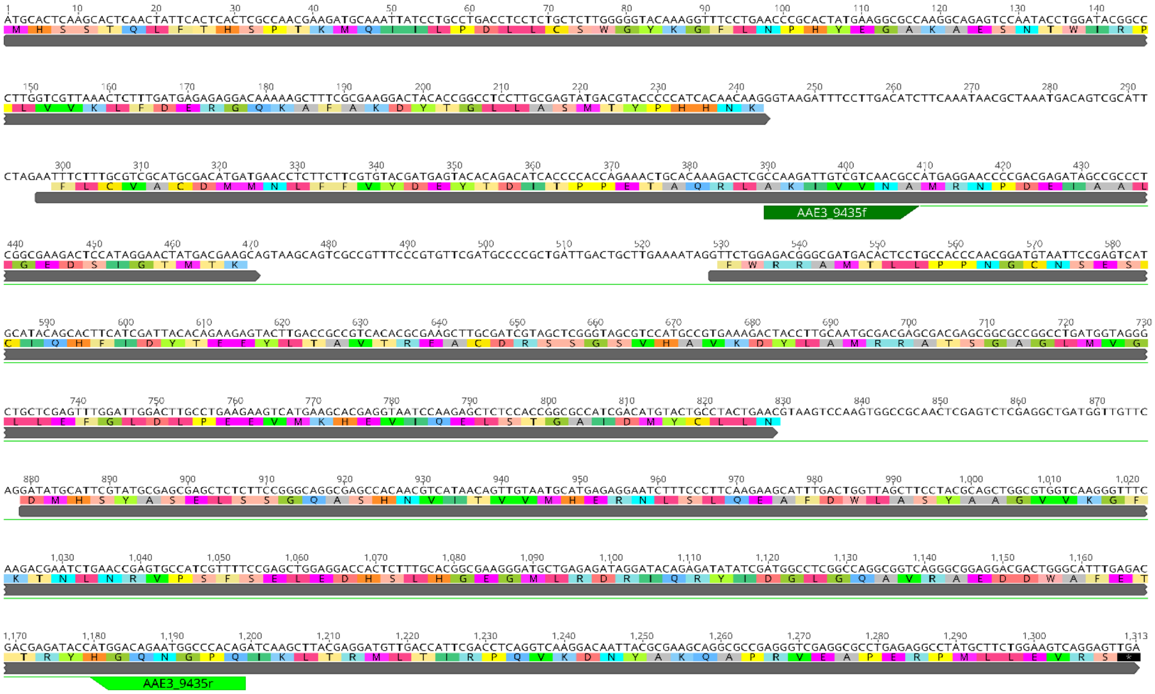

> AAE3_109435 (Aa.LNS)
atgcacagctcgacccagctgttcactcactctccgaccaaaatgcagatcatcctgccggatctgctgtgctcctggggat
ataaagggtttctcaaccctcactatgagggcgctaaggcggaatccaacacctggattcgcccgctcgtagtgaaactttt
cgacgaacgtggccagaaagcgttcgccaaagattacaccggtctgctggcgtctatgacttacccgcaccacaacaaa
gaattcctgtgtgtggcttgcgatatgatgaacctgttctttgtttatgacgagtataccgatattaccccgccggaaaccgcgc
agcgtctggctaaaatcgttgtaaatgctatgcgcaatccggatgagatcgccgctctgggtgaagacagcatcggtactat
gaccaagcagttctggcgccgcgctatgactctgctgccaccgaacggttgtaactctgaatcttgtatccagcacttcattga
ctacaccgaagaatatctgactgcagtgacgcgtgaagcttgtgatcgtagcagcggctctgtccacgctgtaaaagactat
ctggcgatgcgtcgtgcaacctctggcgcgggtctgatggttggtttactggaatttggcctcgatctgccggaagaagtcatg
aaacatgaagtgatccaggaactgtctactggcgcgatcgacatgtactgtctgctgaacgacatgcatagctacgcatctg
aactgtcgtctggtcaggcctctcacaacgttattaccgtcgtgatgcacgaacgtaacctgtccctgcaagaagcttttgatt
ggctggcctcttacgcggcaggcgtagtgaagggtttcaaaaccaacctgaaccgcgttccttcttttagcgaactggagga
ccactctctgcacggcgaaggcatgctccgtgatcgcattcagcgttacatcgacggcctgggtcaggcggttcgcgcgga
agatgattgggcttttgaaactacccgctatcacggtcagaacggcccgcagattaaactgacccgtatgctgaccatccgt
ccgcaggttaaggacaactacgctaaacaggctccgcgcgtcgaggctcctgaacgcccgatgttactggaagtgcgta
gttaa

Fig. S1. Nucleic acid sequence of AAE3_109435 in the genome and codon-optimized nucleic acid sequence for *E. coli*. The two primers (AAE3_9435f and AAE3_9435r) used for PCR amplification were highlighted here.

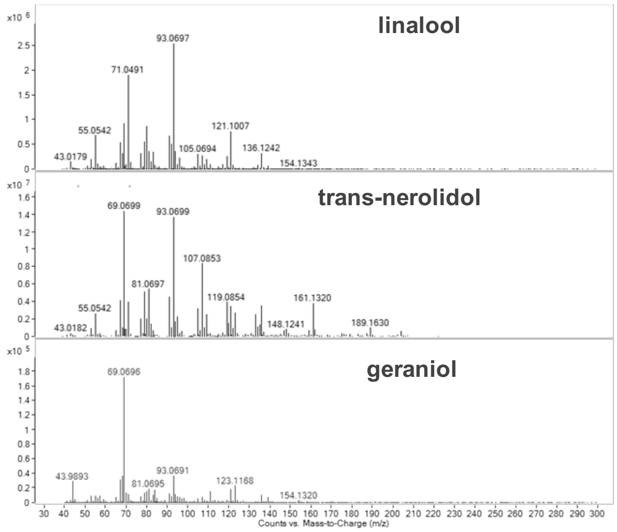

Terpenes	Calculated RI	Literature RI
linalool	1099	1086
nerolidol	1567	1564
geraniol	1259	1256
γ-muurolene	1486	1472
germacrene D	1498	1481
(+)-δ-cadinene	1527	1516

Fig. S2. Mass spectra and retention indices for terpenes detected in this study. Retention indices (RIs) were calculated by calibrating with GC-MS with a C8–C30 alkane mix and compared with literature data in National Institute of Standards and Technology database.

Names in this study	Entry	E-value	Score	Identity	Gene names	Organism
Galma_223690	A0A067THX9	0	1,461	77.20%	GALMADRAFT_223690	Galerina marginata (strain CBS 339.88)
Galma_63556	A0A067T8I8	0	1,324	71.60%	GALMADRAFT_63556	Galerina marginata (strain CBS 339.88)
Hypsu1_148365	A0A0D2NH86	3.10E-171	1,257	65.90%	HYPSUDRAFT_148385	Hypholoma sublateritium FD-334 SS-4
Hypsu1_148385	A0A0D2NA50	1.30E-159	1,180	64.90%	HYPSUDRAFT_148365	Hypholoma sublateritium FD-334 SS-4
Galma_266794	A0A067T571	3.60E-94	750	45.50%	GALMADRAFT_266794	Galerina marginata (strain CBS 339.88)
M413_27416	A0A0C2YLE7	6.90E-92	738	43.10%	M413DRAFT_27416	Hebeloma cylindrosporum h7

```
Agrped1_689675     1    MSSQIYIPDLLITWPWQKVRNPLLQEVQDEANEWVKSFVLFEPEQFEKFKACDFNLLGAL    60
Galma_223690       1    MPSQFTIPDLLITWPWQEITNPMLHEVDAEANEWVQSLNLFEPKQFEKFKACNFNLLGSL    60
                        * **: ***********:: **:*:**: ******:*: ****:********:*****:*

Agrped1_689675     61   VGPLGTKEELRISCDLMNFYFAFDEYTDLASADEAKVIARDVMESFRHTDKPSHNKITEM    120
Galma_223690       61   VGPLPSRDHLRVSCDLMNFYFAFDEYTDMANKDEAMRIARDVMQAFRNTDTPSNSKITEM    120
                        **** :::.**:****************:*. ***  ******::**:**.**:.*****

Agrped1_689675     121  ARQFFERTINTVGNDPTGIEQFIADFDAYTTSIIQEADDRASGHIRSVEDYFILRRDTCG    180
Galma_223690       121  ARQFFKRTIEVVGEDLPGIERFIADFDAYTRSVIQEADDRVAGHIRNVEDYFILRRDTCG    180
                        *****:***:.**:*  ***:********* *:*******.:****.*************

Agrped1_689675     181  GKPSFSFFGLGLNIPKEVFAHPMFISMTESATDLIAITNDMHSYNLEQSRGLDGHNVITA    240
Galma_223690       181  AKPSFSFYGLGLNIPTEVFEHPLLISMVESATDLIAVTNDMHSYGLEHSRGLDGHNVITA    240
                        .******:*******.*** **::***.********:*******.**:************

Agrped1_689675     241  IMHEYKINLQGALYWLSGYATKTIAKFISDRKNLPSWGPVVDRAVEQYFDRVGRCVRGYD    300
Galma_223690       241  IMHEYQLDLQGALYWLSGYATKTISKFLTDRKNLPSWGPTIDKALEIYLDRLGRCVRGYD    300
                        *****:::****************:**::**********.:*:*:* *:**:********

Agrped1_689675     301  AWSYETKRYYGKNGLEIQKTRQITLRPLDPAYVTKEQLQVSMKA                    344
Galma_223690       301  AWSYSTKRYYGKNGLKVQKTRRITLKPRDAAYITKDQLQVSIA-                    343
                        ****.**********::****:***:* * **:**:*****:
```

Fig. S3. The alignment between Agrped_689675 and Galma_223690 and BLAST search results in UniProt database with Agrped_689675 (or Ap.LS). The search was done in March-2018 with the top 6 proteins listed here. The results may change today as new proteins are being deposited into UniProt database.

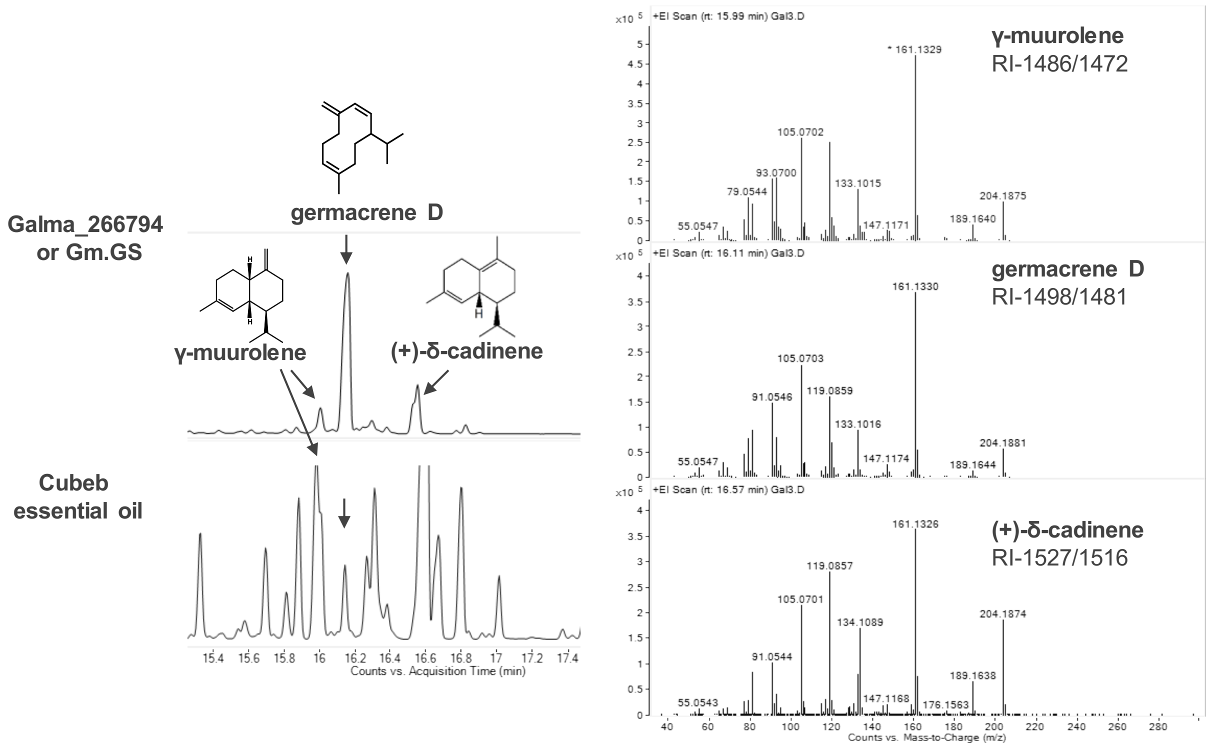

Fig. S4. GCMS chromatograms and spectra for Galma_266794. Retention indices (RIs) were listed with the calculated values on the left and literature values on the right. Germacrene D and γ-muurolene are further verified by Cubeb essential oil. Cedrela woods oil was used as the authentic standard of (+)-δ-cadinene [1].

1. Aa.LS (fungal)
2. Sc.LNS, D5SL78(bacterial)
3. Zm.LNS, Q29VN2 (plant)
4. Ma.LS, Q8H2B4 (plant)

	1	2	3	4
1		15.2%	13.4%	10.0%
2	15.2%		7.8%	10.4%
3	13.4%	7.8%		24.0%
4	10.0%	10.4%	24.0%	

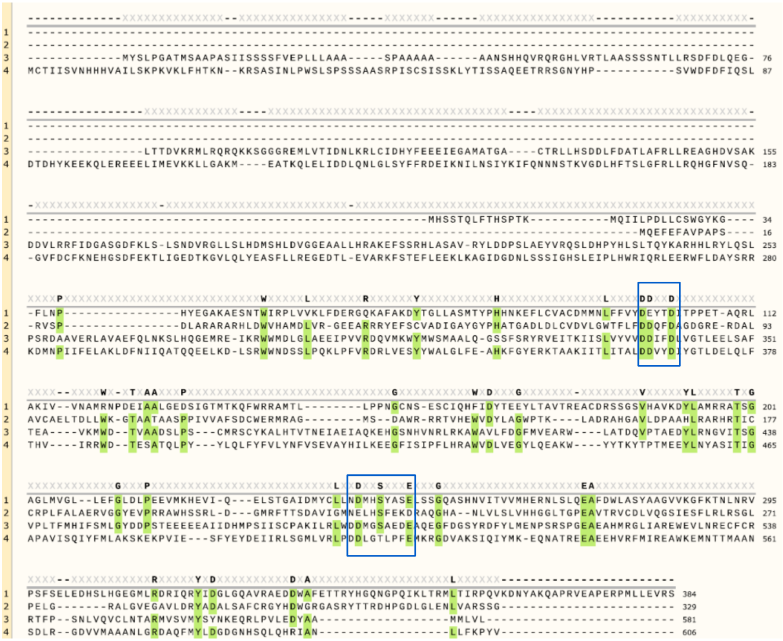

Fig. S5. Amino acid sequence alignment and identity table of LSs and LNSs from fungi, bacteria and plants. The two conserved regions, aspartate-rich motif and NSE triad were highlighted in blue boxes. The sequence identity table indicated the large difference of LSs among different kingdoms. The overall sequence identity is very low, and the fungal LS is slightly more similar to the bacterial LNS than to the plant LS and LNS. Alignment was done with Clustal Omega program v1.2.4.

A

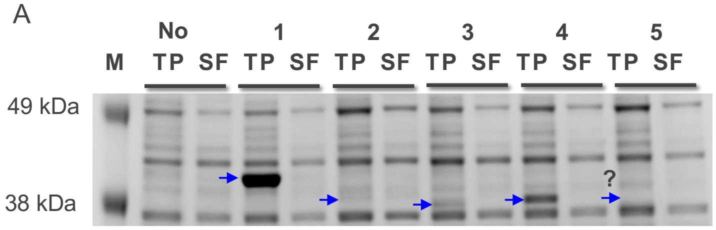

No.	Enzymes	kDa
1	AAE3_109435, Aa.LNS	43.4
2	Agrped1_689675, Ap.LS	39.7
3	Agrped1_689671, Ap.LNS	39.8
4	Galma_223690, Gm.LNS	39.4
5	Hypsu_148385, Hs.LNS	39.2

B

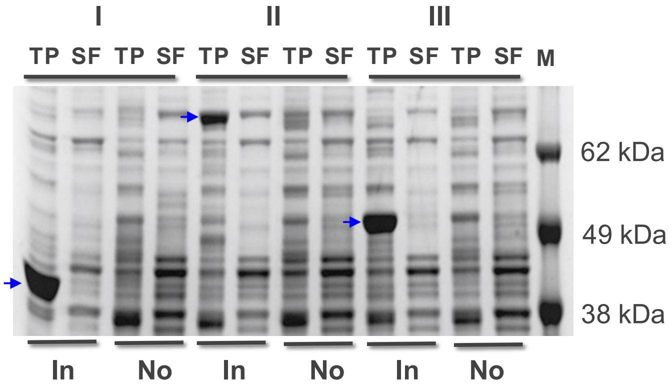

No.	Enzymes	kDa
I	Aa.LNS	43.4
II	MBP_Aa.LNS	85
III	TrxA_Aa.LNS	57

M – marker
TP – total protein
SF – Soluble fraction
In – induced
No – not induced, as control

Fig. S6. (A) Expression and solubility analysis of the fungal LNSs and LS. (B) Solubility analysis for the fusion Aa.LNS with maltose binding protein (MBP) or thioredoxin (TrxA). M: protein ladder, TP: total proteins, SF: soluble fraction, In: induced cells, No: not induced as control.

9435.tf16.BL21 at different Arabinose and IPTG concentrations

Chaperone	/	pGro7		pTf16												
IPTG (mM)	/	0	0.1	0	0.1						0.2					
Arabinose (mM)		0	10	0	1.1		3.3		10		1.1		3.3		10	
	M	TP	In	No	TP	SF	TP	SF	TP	SF	TP	SF	TP	SF	TP	SF

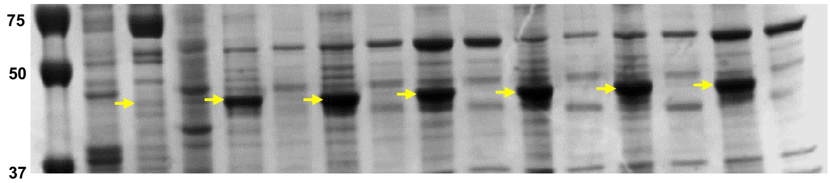

Chaperone	/	pGro7		pTf16												
IPTG (mM)	/	0	0.1	0	0.4						0.8					
Arabinose (mM)		0	10	0	1.1		3.3		10		1.1		3.3		10	
	M	TP	In	No	TP	SF	TP	SF	TP	SF	TP	SF	TP	SF	TP	SF

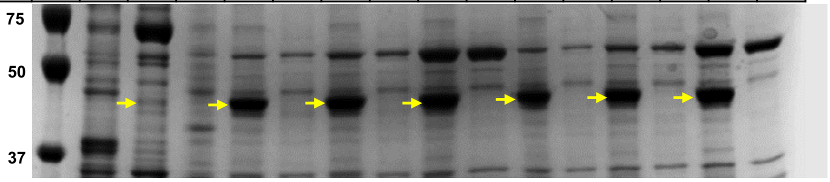

M - marker
TP - total protein
SF - soluble fraction
In – induced
No – not induced

Fig. S7. The optimization of Aa.LNS solubility with the co-expression of chaperone proteins. Two chaperone systems were used here: pGro7 expressing groES-groEL proteins, pTf16 expressing the trigger factor protein (tig). IPTG and arabinose were used to induce the expression of Aa.LNS and chaperones, respectively. It was found arabinose concentration (or chaperone expression levels) has invisible effect on the solubility of Aa.LNS. Hence, 3.3mM arabinose and 0.1 mM IPTG were used for the large-scale expression and purification experiments. M: protein ladder, TP: total proteins, SF: soluble fraction, In: induced, No: not induced as control.

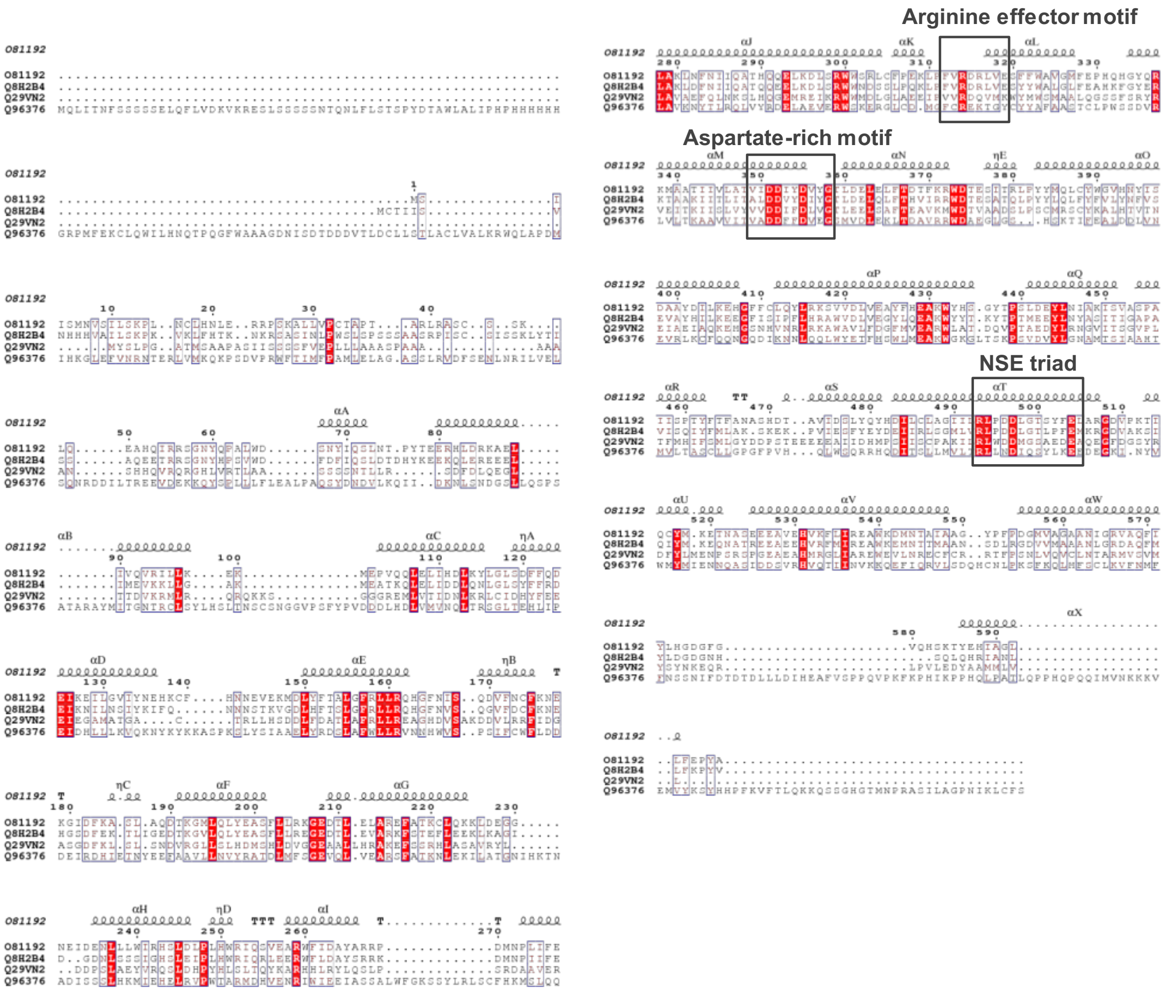

Fig. S8. The sequence alignment and their secondary structures of plant terpene synthases. The three regions, arginine effector motif, aspartate-rich motif and NSE triad, were highlighted in black boxes. The four proteins were (1) O81192, PDB ID 1n1b/1n24, (+)-bornyl diphosphate synthase from *Salvia officinalis*; (2) Q8H2B4, (*R*)-linalool synthase from *Mentha aquatica* (Water mint); (3) Q29VN2, linalool/nerolidol synthase from *Zea mays* (Maize); (4) Q96376, S-linalool synthase from *Clarkia breweri* (Fairy fans). The figure was prepared with the ESPript 3.0 [2].

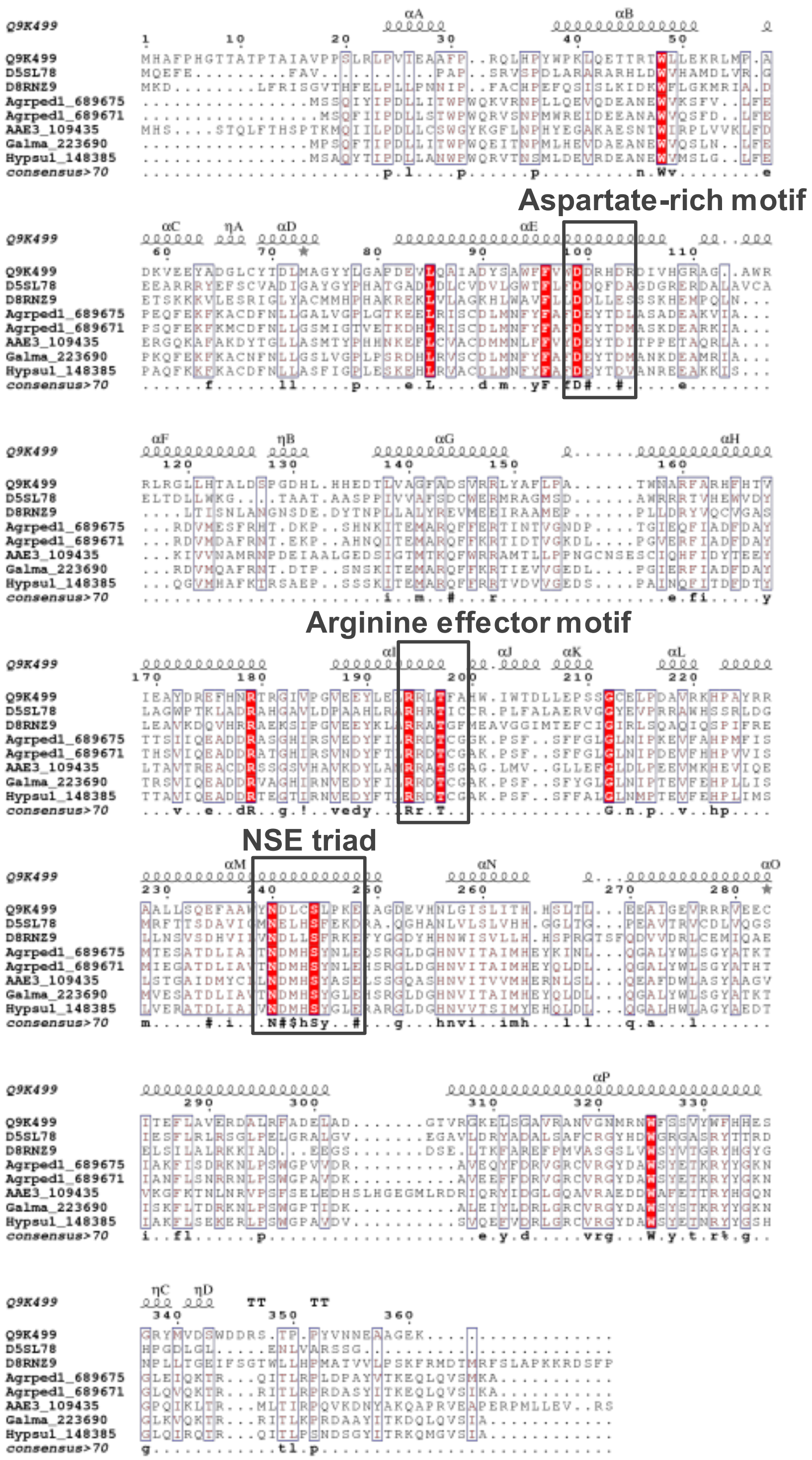

Fig. S9. The sequence alignment and their secondary structures of microbial terpene synthases. The three regions, arginine effector motif, aspartate-rich motif and NSE triad, were highlighted in black boxes. The eight proteins were (1) Q9K499 (PDB ID 4LXW), Epi-isozizaene synthase from *Streptomyces coelicolor*; (2) D5SL78, Sc.LNS from *S. clavuligerus*; (3) D8RNZ9, LNS from Spikemoss; (4) Ap.LS; (5) Ap.LNS; (6) Aa.LNS (7) Gm.LNS; (8) Hs.LNS. The figure was prepared with the ESPript 3.0 [2].

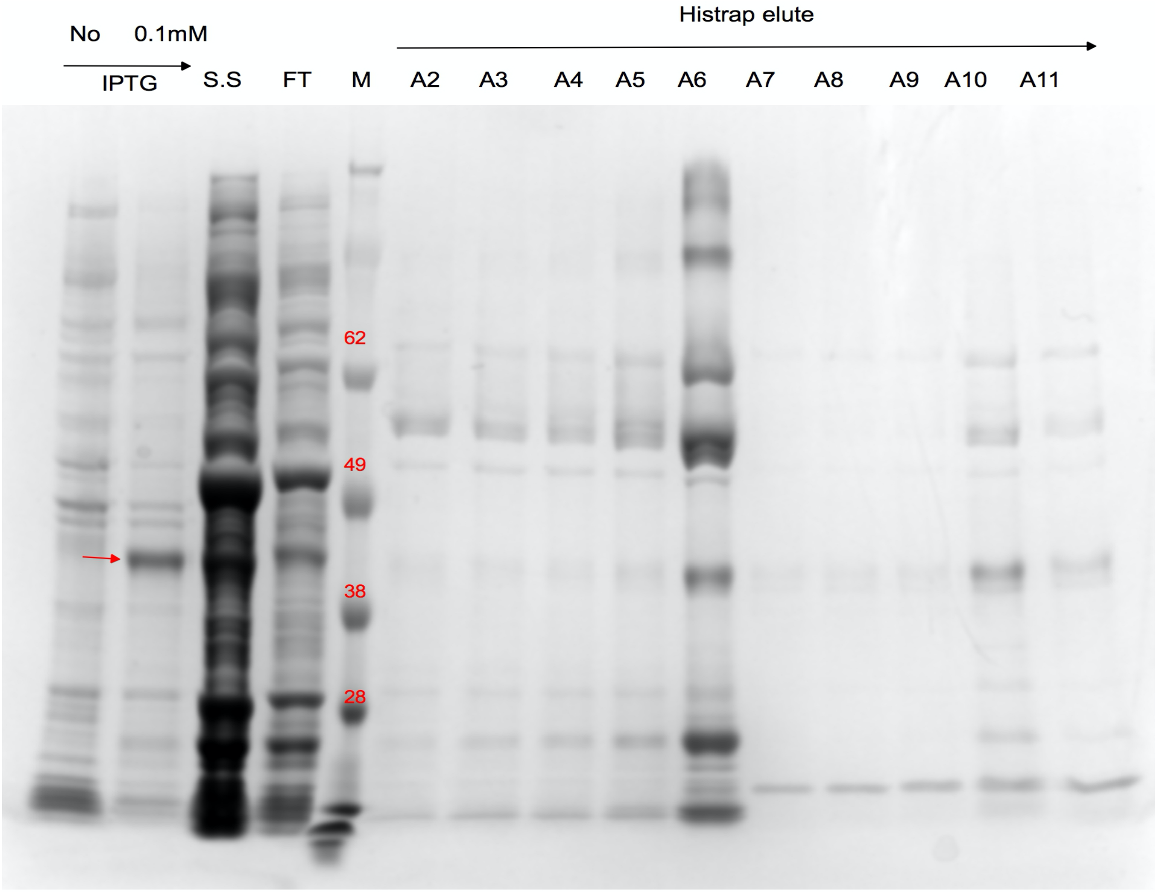

S.S = Supernatant after sonication
FT = Flowthrough after HisTrap binding

Fig. S10. Purification of Aa.LNS, full image for Fig. 4B.

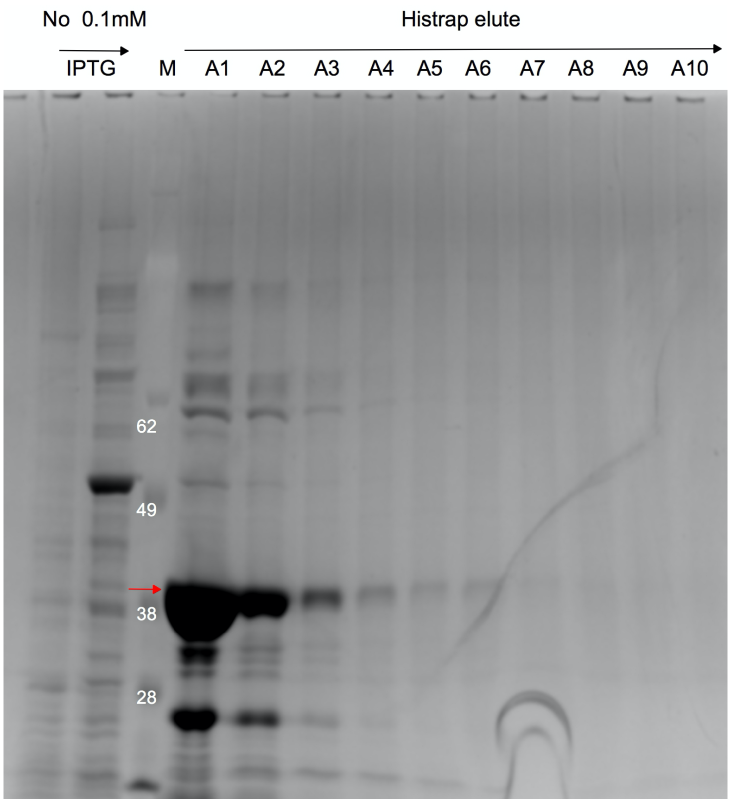

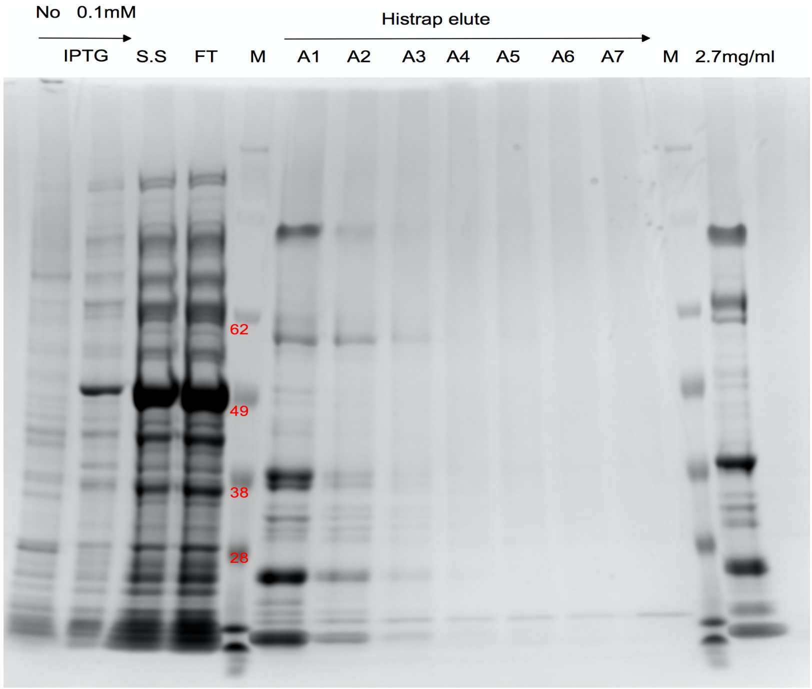

S.S = Supernatant after sonication
FT = Flowthrough after HisTrap binding

Fig. S11. Purification of Ap.LS, full image for Fig. 4A.

Reference

1. Agger, S., Lopez-Gallego, F. & Schmidt-Dannert, C. Diversity of sesquiterpene synthases in the basidiomycete *Coprinus cinereus*. *Molecular microbiology* **72**, 1181-1195 (2009).
2. Robert, X. & Gouet, P. Deciphering key features in protein structures with the new ENDscript server. *Nucleic Acids Research* **42**, W320-W324 (2014).
