# Supplementary material for: Bioinformatics-aided identification, characterization and applications of mushroom linalool synthases
Source: Commun Biol. 2021 Feb 17;4:223. doi: 10.1038/s42003-021-01715-z (PMC7890063; doi:10.1038/s42003-021-01715-z)
Supplement: Supplementary file 2 — Description of Additional Supplementary Files [file 42003_2021_1715_MOESM2_ESM.pdf]

Description of Additional Supplementary Files

File name: Supplementary Data 1
Description: Source data (protein sequence fasta file) in Figures 3A.

File name: Supplementary Data 2
Description: Source data in Figures 4A and B.

File name: Supplementary Data 3
Description: Source data in Figures 4C and D 2 (Linalool yield comparison of different linalool
synthases).
